# Parametrization of β^3^‑Peptides for Coarse-Grained Molecular Dynamics Simulations

**DOI:** 10.1021/acs.jcim.5c03108

**Published:** 2026-03-20

**Authors:** O. Pavela, A. Wacha, T. Beke-Somfai, A. K. Sieradzan

**Affiliations:** † Institute of Materials and Environmental Chemistry, 557117 HUN-REN Research Centre for Natural Sciences, Magyar tudósok körútja 2, Budapest H-1117, Hungary; ‡ Faculty of Chemistry, Laboratory of Molecular Modeling, 49646 University of Gdańsk, ul. Wita Stwosza 63, Gdańsk 80-380, Poland; § Hevesy György Ph.D. School of Chemistry, Eötvös Loránd University, Budapest H-1117, Hungary

## Abstract

Coarse-grained simulations of foldamers such as β-peptides require force fields that accurately capture the backbone geometry and flexibility. In this work, we extend the UNRES coarse-grained model to β^3^-peptides by reparameterizing key local potential terms: virtual-bond stretching, virtual bond-angle bending, and torsional potentials. The bond-stretching term was derived from probability distributions obtained via all-atom molecular dynamics simulations of a reference β-peptide, while the angular and torsional potentials were fitted to quantum chemical potential energy surfaces computed by using the GFN2-xTB method with implicit solvent. Analytical potential forms were used to model the energy landscapes, and coefficients were obtained via nonlinear fitting to the potential of mean forces (PMFs). The modified UNRES model was validated through coarse-grained simulations and compared to the all-atom reference in terms of structural properties such as radius of gyration, end-to-end distances, and intramolecular side-chain separations. The capacity of the extended force field to reproduce β-peptide helical conformations was also evaluated with a peptide. Furthermore, the ability of the model to reproduce peptide self-assembly was evaluated using two peptides, one that is known to form large aggregates in aqueous solution and another that does not. The simulations successfully recapitulated these experimentally observed behaviors. Overall, the results demonstrate that the newly derived local potentials for β-amino acids can capture overall peptide behavior, making the model suitable for predictive simulations of β-peptide folding and aggregation.

## Graphical abstract

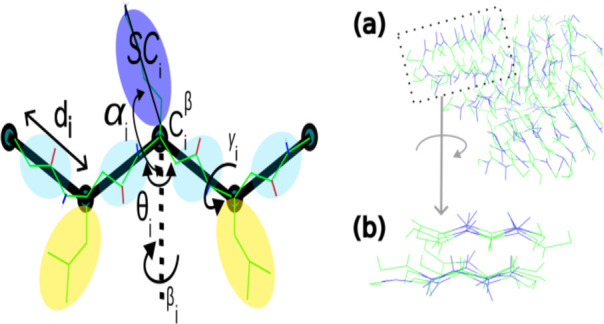

## 1 Introduction

In the last three decades a diverse use of β-peptides can be seen in diverse research areas from nanostructures in materials science,^1–4^ through biocatalysis to biomedical applications such as receptor agonist or novel antibiotics.^5^ Moreover, β-peptides have been developed for other purposes as well, such as cholesterol absorption inhibition,^6^ somatostatin receptor agonist,^7^ hDM2 inhibition.^8^ The search for new antibiotics is motivated by the widespread overuse of conventional antibiotics which has led to the emergence of multidrug-resistant pathogens, rendering many standard treatment strategies increasingly ineffective.^9–11^ In recognition of this growing threat, the World Health Organization (WHO) has identified antimicrobial resistance (AMR) as one of the most pressing global health challenges,^12–14^ which now stimulates intensive research focusing on various compounds that could provide solutions against AMR. Among these, antimicrobial peptides (AMPs) which are natural peptides produced as part of their innate immune defense system, exhibit broad-spectrum antimicrobial activity.^15,16^ However, one disadvantage of these natural peptides is their low biostability,^17,18^ which limits widespread therapeutic use. To overcome this limitation, non-natural compounds have been synthesized which can withstand proteolytic degradation.^19–21^ Among these, non-natural *beta*-peptides (peptides consisting β-amino acids) have been used to develop compounds with antimicrobial activity.^22–24^ Note that as a result of their extra methylene group, the orientation of their side chains needs to be controlled to achieve certain structures (Figure 1). Moreover, these compounds sometimes exhibit a high self- or co-assemble affinity, and thus they can obtain helical bundles,^25,26^ protein-like assemblies,^27,28^ and nanofibers.^29,30^ Considering challenges of experimental techniques to identify structural details of the β-peptides higher ordered structures, theoretical simulations with molecular dynamics (MD) simulations often provide detailed insight as an alternative to experiments.^31,32^ However, assembly formation is a crucial part of the aggregation process, which is a major challenge for MD due to the complex interactions, the number of participating molecules, and the time scales on which self-assembly occurs. With the slow kinetics of these processes,^33^ coarse-grained (CG) molecular dynamics (MD) simulations are often preferred over all-atom (AA) simulations for studying large-scale peptide aggregation. In CG models, groups of approximately 4–6 heavy atoms are represented by a single particle (bead), which substantially reduces the number of particles in the system and allows for the simulations of larger molecular assemblies over extended time scales using comparable computational resources. As a result, CG MD is well suited for exploring mesoscale phenomena, such as peptide self-assembly. Self-assembly of α-amino acid containing peptides have been computed with the UNRES^34,35^ and MARTINI^36,37^ CG force fields for years. However, for β-amino acid containing peptides, there are no coarse-grained molecular dynamics force fields, which can model them. As part of earlier efforts for understanding β-peptide self-assembly, software support for their quick buildup,^38^ and MD parameters have been developed,^39^ where our group used a torsional minimum energy path matching on quantum chemical results which was implemented to the CHARMM36m^39^ force field to allow accurate reproduction of assembly processes.^22,40^ To progress further, in this work, we modified the UNRES force field to simulate β-peptides, more specifically β^3^-peptides, and tested the parameters obtained mainly with two β^3^-peptides, which will be referred to as 3K and EK (EK is also called as BUTLEU), for evaluating secondary structure features, we used a peptide, referred to as PeptideIV, which was reported to form stable helical structure in water.^41,42^ Figure 2 shows the structures of all three β^3^-peptides. We modified the local potential terms in the UNRES force field. The bond-stretching term was calculated from the probability distribution obtained from all-atom simulation. The angle-bending and torsional potentials were derived from quantum chemical potential energy surfaces, computed using the GFN2-xTB method^43^ with implicit solvent. The test simulations with 3K, EK monomers, and multimers were compared to their equivalent all-atom simulations as well as to experimental data.

**Figure 1:**
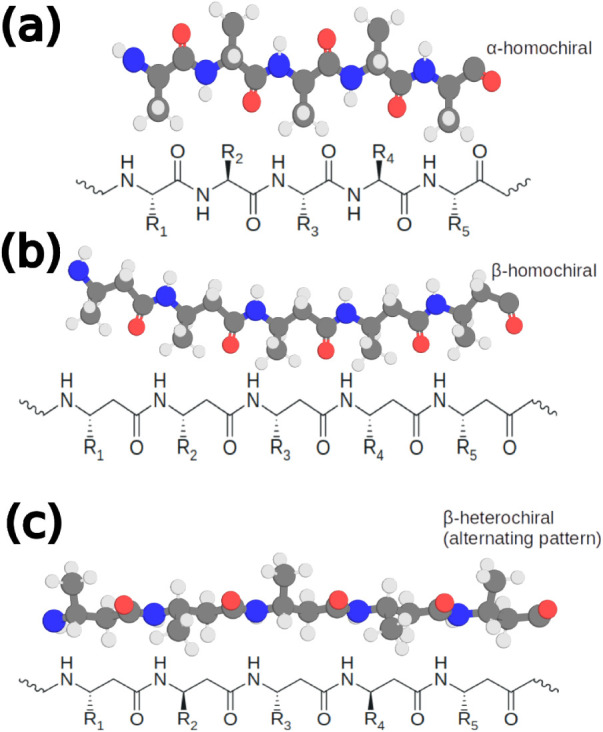
Schematic representation of the side-chain patterns in extended homochiral α- and β-peptides (panels (a) and (b), respectively), and alternating heterochiral β-peptides (panel (c)). The lower part of each panel displays the chemical structures of the theoretical sequences, while the upper part presents a ball-and-stick view illustrating the spatial arrangement of side chains along the extended backbone, using alanine residues as an example (color scheme: carbon—gray, nitrogen—blue, oxygen—red, hydrogen-white). In the heterochiral β-peptide sequences, the chirality at the β^3^ carbon alternates between the R and S configurations across the chain.

**Figure 2:**
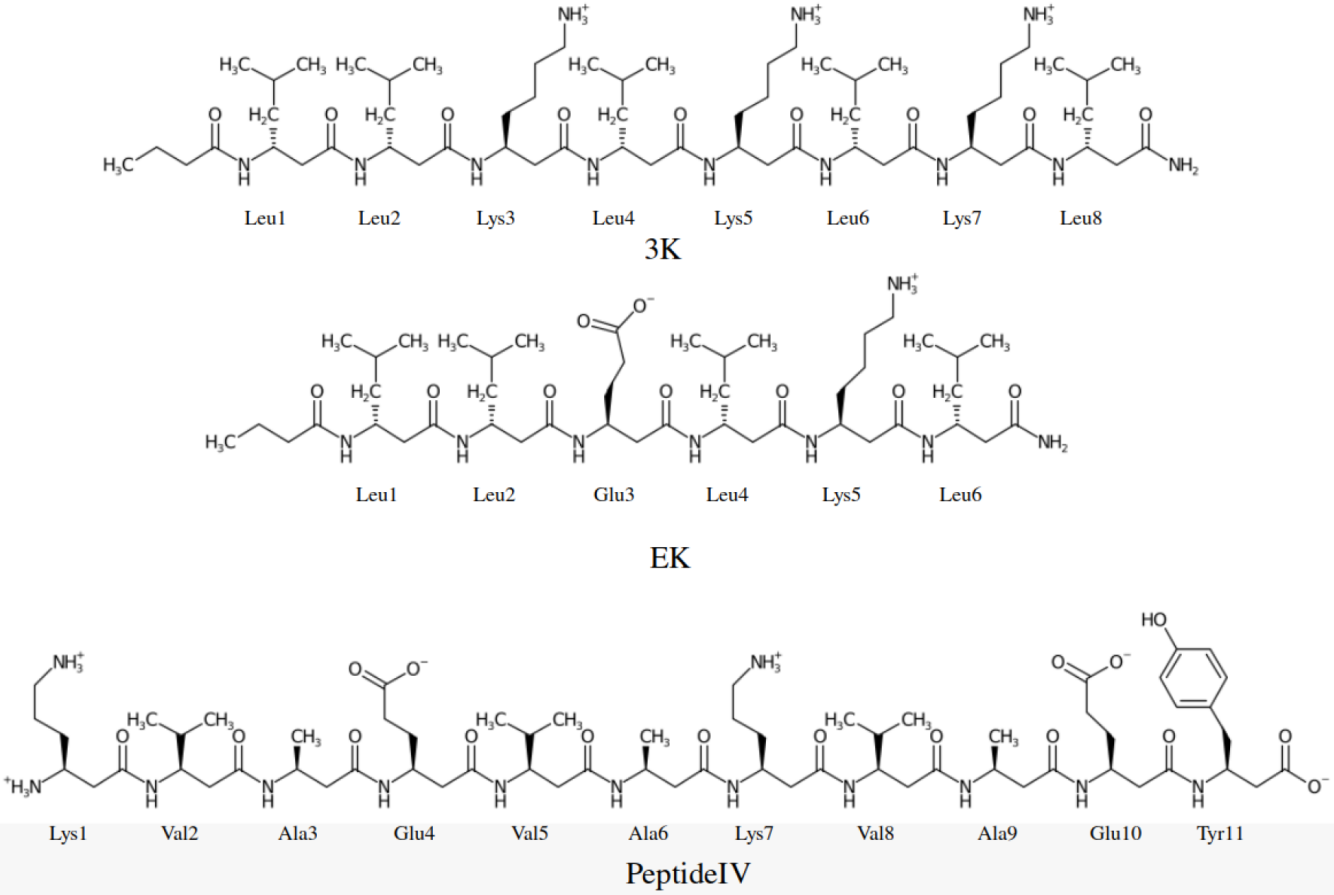
Structure of 3K, EK, and PeptideIV.

## 2 Methods

We employed a strategy analogous to that of A. Liwo et al.^44^ for the parametrization of β-amino acids, specifically in the derivation of virtual-bond-angle bending and torsional potentials. In that approach, backbone-local virtual-bond-angle and torsional potentials are derived by mapping ab initio energy surfaces of effective potentials that reproduce the underlying quantum-mechanical energetics with use of the Levenberg–Marquardt algorithm. The bond-stretching potential was derived and fitted using data obtained from all-atom molecular dynamics simulations of a representative β-peptide.^22^ We decided not to apply a quantum-mechanical approach for this term, as it is a simple harmonic potential and only the spring constant and the equilibrium distance had to be determined. In principle, the angle bending and torsional terms could be derived from all-atom simulations of single, blocked β-peptides as well; however, they cannot be derived from long peptides, as the UNRES formalism does not apply typical torsional terms but rather the torsional-valence terms and derives them from primitives. The simulation in QM, as in previous papers of A. Sieradzan et al., seemed to be a force field-consistent approach.^44,45^ The nonbonded terms have not been modified, as the U_*SCiPj*_ potential is a volume-excluded potential, whereas the main contribution of U_*PiPj*_ in the UNRES force field arises from the dipole–dipole interactions. As β-peptides peptide groups, mainly in approximation, are spheroids of revolution along the longer axis, the dipole–dipole character is maintained; therefore, we assumed a similar interaction strength as in the case of α-peptide peptide groups. One could expect the larger size of the β-amino acid peptide group with the β-carbon atom than the peptide in α-peptide; however, the main difference is in the size of the longer axis in the ellipsoid of revolution, not in the shorter (perpendicular). Therefore, the short axis should remain the same, and anisotropy should change. However, as in the current version, peptide groups are modeled as spheres, not ellipsoids of revolution. The change in size would lead to too large distances between chains of aggregated β-peptides; therefore, we maintained the U_*SCiPj*_ or U_*PiPj*_, as in the original UNRES. Lastly, parametrization work for charges was not needed, as in the current version of UNRES, no explicit charges are used. Side-chain–side-chain interactions are modeled by the Gay–Berne potential, whereas peptide-group–peptide-group interactions are modeled via dipole–dipole interactions, with parameters stored in the force field; thus, no additional charge parametrization was required.

### 2.1 UNRES

In the UNRES model, polypeptides composed of α-amino acids are coarse-grained by representing each residue with an α-carbon (Cα) atom, a united side chain (SC), and a united peptide group placed midway between adjacent Cα atoms. Only the side chains and peptide groups serve as interaction sites, while the Cα atoms define the overall geometry of the backbone. To adapt this model for β^3^-amino acids, we substituted the α-carbon atoms with β-carbon (Cβ) atoms, which represent the backbone β^3^ carbon atoms. The coarse-grained degrees of freedom consist of Cβ-Cβ and Cβ-SC virtual bond vectors. These can alternatively be described by using virtual bond angles (θ), dihedral angles (γ), and orientation angles (α_SC_, β_SC_) that define the spatial arrangement of the SC center relative to the backbone.

The energy function of UNRES is given by eq 1. It consists of the long-range intersite terms: side-chain–side-chain, side-chain–peptide group, and peptide-group–peptide interactions ( $U_{SC_{i}SC_{j}}$ , $U_{SC_{i}p_{j}}$ , and $U_{p_{i}p_{j}}$ ); the local terms that, in turn, comprise the virtual-bond-deformation (*U*_*bond*_), virtual-bond-angle terms (*U*_*b*_), the backbone-torsional (*U*_*tor*_), and the side-chain-rotamer terms (*U*_*rot*_), the third-order multibody terms ( $U_{corr}^{\left(3\right)}$ and $U_{turn}^{\left(3\right)}$ ); these terms account for the coupling between the backbone-local and backbone-electrostatic interactions 1,2), and the disulfide-bond potentials (*U*_*ssbond*_) describing disulfide bond formation/breaking. Each energy term is multiplied by an appropriate weight (ω) (Table S1 shows the exact parameter values); higher-order terms are also multiplied by a temperature factor as described in earlier work.^46^ In this work, we modified three local potentials—*U*_*bond*_, *U*_*b*_, and *U*_*tor*_—to adapt the UNRES model for β^3^-amino acids. The side-chain rotamer terms remained unchanged. The full energy expression is given in eq 1, and the coarse-grained variables are illustrated in Figure 3. It should be noted that we adopted the UNRES model straightforwardly, which indicates that simulation of an “all-L” amino acid sequence in UNRES notation is in fact an alternating D- and L-amino acid sequence for β-amino acids.

**Figure 3:**
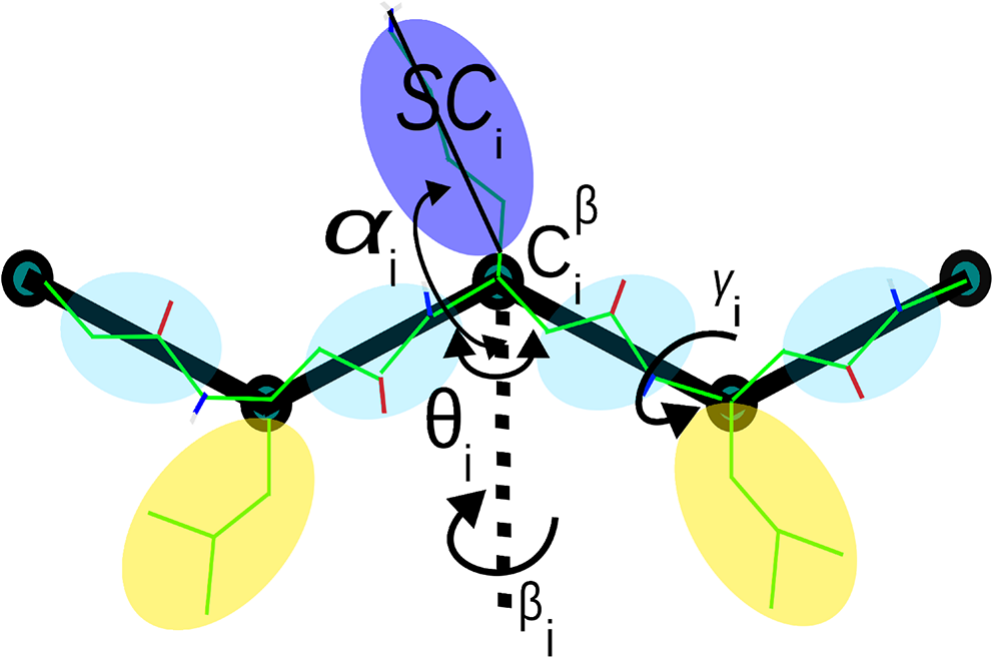
UNRES model of polypeptide chains applied for β^3^ peptides. The interaction sites are united peptide groups located between consecutive β-carbon atoms (light-blue spheres) and united side chains attached to the β-carbon atoms (spheroids of various colors and dimensions). The backbone geometry of the simplified polypeptide chain is defined by the Cβ-Cβ-Cβ virtual-bond angles θ (with θ*_i_* having the vertex at the central $C_{i}^{\beta }$ ), and the Cβ-Cβ-Cβ-Cβ virtual-bond dihedral angles γ*_i_* (with γ*_i_* having the axis passing through $C_{i}^{\beta }$ and $C_{i+1}^{\beta }$ ). The local geometry of the *i*th side-chain center is characterized by the polar angle α*_i_* (the angle between the bisector of the corresponding θ*_i_* angle and the $C_{i}^{\beta }-{\mathrm{SC}}_{i}$ vector), and the azimuthal angle β*_i_* (the angle of counterclockwise rotation of the $C_{i}^{\beta }-{\mathrm{SC}}_{i}$ vector about the bisector vector lying in the $C_{i-1}^{\beta }-C_{i}^{\beta }-C_{i+1}^{\beta }$ plane, starting from $C_{i-1}^{\beta }$ ). For illustration purposes, all-atom bonds, except for those involving hydrogen atoms attached to carbon, are superposed on the coarse-grained representation.

1 $$\begin{array}{ll} U= & \omega_{SC}\underset{i<j}{\sum }U_{SC_{i}SC_{j}}+\omega_{SCp}\underset{i\neq j}{\sum }U_{SC_{i}p_{j}}+\omega_{pp}^{VDW}\underset{i<j-1}{\sum }U_{p_{i}p_{j}}\\ & +\omega_{pp}^{el}f_{2}\left(T\right)\underset{i<j-1}{\sum }U_{p_{i}p_{j}}^{el}+\omega_{tor}f_{2}\left(T\right)\underset{i}{\sum }U_{tor}\left(\gamma_{i},\theta_{i},\theta_{i+1}\right)\\ & +\omega_{b}\underset{i}{\sum }U_{b}\left(\theta_{i}\right)+\omega_{rot}\underset{i}{\sum }U_{rot}\left(\theta_{i},\alpha_{SC_{i}},\beta_{SC_{i}}\right)+\omega_{bond}\underset{i}{\sum }U_{bond}\left(d_{i}\right)\\ & +\omega_{ssbond}\underset{nss}{\sum }U_{ssbond}\left(dss\right)+\omega_{corr}^{\left(3\right)}f_{3}\left(T\right)U_{corr}^{\left(3\right)}+\omega_{turn}^{\left(3\right)}f_{3}\left(T\right)U_{turn}^{\left(3\right)} \end{array}$$

### 2.2 Potential Energy Surface Computation

We calculated the energy surface for a terminally blocked β-amino acid residue in the angles for rotation of the peptide groups about the Cβ-Cβ virtual-bond axes (Figure 4). Furthermore, we calculated the virtual angle arising between three neighboring Cβs. The terminally blocked β-amino acid side chain was β^3^-homoalanine, which represents every residue that is not glycine or not proline. The amino acid termini were capped with a methyl group at the N-terminus and an ethyl group at the C-terminus to maintain methodological consistency with previous work.^44^

**Figure 4:**
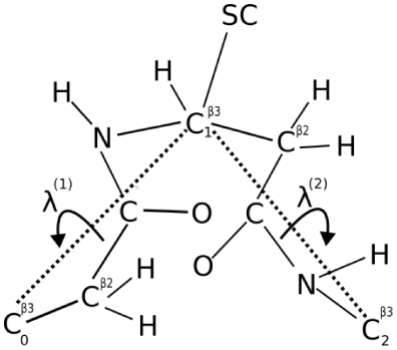
Definition of the λ^(1)^ and λ^(2)^ angles for the rotation of the peptide groups about the C^β3^-C^β3^ virtual-bond axes. λ^(1)^ is the angle for the counterclockwise rotation of the peptide group located between $C_{0}^{\beta 3}$ and $C_{1}^{\beta 3}$ ; λ^(1)^ = 0 when the carbonyl carbon atom of the peptide group is in the $C_{0}^{\beta 3}-C_{1}^{\beta 3}-C_{2}^{\beta 3}$ plane and faces $C_{2}^{\beta 3}$ . λ^(2)^ is the angle for the counterclockwise rotation of the peptide group located between $C_{1}^{\beta 3}$ and $C_{2}^{\beta 3}$ ; λ^(2)^ = 0 when the amide nitrogen atom of the peptide group is in the $C_{0}^{\beta 3}-C_{1}^{\beta 3}-C_{2}^{\beta 3}$ plane and faces $C_{0}^{\beta 3}$ .

The potential energy surfaces (PESs) of terminally blocked β^3^-homoalanine are needed for the construction of the PMFs. We used the GFN2-xTB method with the implicit water solvent model GBSA as implemented in the xtb program (version 6.7.1), developed by Grimme and coworkers.^43^ Implicit solvation was used to maintain consistency with the implicit-solvent representation of UNRES, for which the effective energy terms are parametrized. The PESs were constructed by minimizing the energy subject to fixed angles λ^(1)^ and λ^(2)^ for the rotation of the peptide groups about the Cβ-Cβ virtual-bond axes, and the virtual-bond angle θ. The grid in angles λ ranged from −180° to 180° with a 15° step size. For each grid point, an additional energy-minimized scan was performed on a grid in θ ranging from θ – 15° to θ + 15°, with a step of δθ = 5°, where θ is the energy-minimized value of θ for λ^(1)^ and λ^(2)^ corresponding to the respective grid point.

The resulting PES values were used to construct a three-dimensional potential of mean force (PMF) surface, representing the effective free energy as a function of λ^(1)^, λ^(2)^, and θ.

### 2.3 Fitting of Analytical Functions to Obtained Potential Energy Surfaces

To obtain force field parameters, we fitted the analytical functions to the PMF computed from the PES. In the following sections, the fitting of parameters to each energy term modified is described.

#### 2.3.1 Bond-Stretching Potential (*U*_*bond*_)

To parametrize the bond-stretching term, we analyzed a 500 ns all-atom molecular dynamics simulation of a reference β-peptide called 3K; the details of this simulation are described later in Section 2.4. The distribution of distances between neighboring β-carbon atoms (Cβ) was calculated. Then a one-dimensional potential of mean force (PMF) was constructed from the normalized histogram of these distances. The PMF is defined as eq 2.

2 PMF(x)=−RTln⁡P(x)

where *P*(*x*) is the normalized probability of observing bond length *x*, obtained from the histogram, *R* is the gas constant, and *T* is the absolute temperature. The values needed for the harmonic fit (eq 3) were determined based on the initial PMF curve. We then fitted a harmonic function to the PMF minimum using

3 $$\omega_{bond}=\frac{1}{2}k{\left(x-x_{0}\right)}^{2}$$

Here, *x*_0_ is the most probable Cβ-Cβ distance from the distribution histogram, and *k* is the curvature at the minimum. The force constant *k* was estimated using a second-order finite difference approximation based on the PMF values at two points adjacent to *x*_0_.

#### 2.3.2 Virtual Bond Angle Bending Potential (*U_b_*)

The potential governing the backbone virtual-bond angle θ was derived from the previously mentioned quantum chemical energy scans that were performed.

For each fixed value of θ, energy minimizations were carried out over the remaining degrees of freedom. The angle θ was sampled around its minimum value in steps of δθ = 5° over a ±15° range. The resulting potential energy values were used to build the PMF according to eq 4, and then we fit using a truncated Fourier series with 12 terms (eq 5).

4 $$PMF\left(\theta \right)=-RT\ln\left(\underset{\lambda^{\left(1\right)},\lambda^{\left(2\right)}}{\sum }e^{-E(\lambda^{\left(1\right)},\lambda^{\left(2\right)},\theta )/RT}\right)$$

where *R* is the gas constant, and *T* is the temperature (298 K).

5 $$U_{bX}\left(\theta \right)=\overset{12}{\underset{i=0}{\sum }}a_{x}^{n}\cos\left(n\theta \right)$$

where $a_{x}^{n}$ are expansion coefficients obtained by least-squares fitting.

#### 2.3.3 Torsional Potential (*U*_*tor*_)

As described previously, we scanned the PES of a protected β^3^-homoalanine as a function of the virtual dihedral angles λ^(1)^ and λ^(2)^, corresponding to the rotations of peptide groups about adjacent Cβ-Cβ axes.

The resulting 3D PES was used to calculate the PMF values and obtain PMF surfaces. The PMF surface was fit using the analytical form given (eq 6).

6 $$PMF\left(\theta_{i},\theta_{j},\gamma_{k}\right)=-RT\ln\left[\underset{\lambda^{\left(1\right)},\lambda^{\left(2\right)},\lambda^{\left(3\right)}}{\sum }\exp\left(\frac{-E_{1}\left(\theta_{i},\lambda^{\left(1\right)},\gamma_{k}-\pi -\lambda^{\left(2\right)}\right)-E_{2}\left(\theta_{j},\lambda^{\left(2\right)},\lambda^{\left(3\right)}\right)}{RT}\right)\right]-PMF\left(\theta_{i}\right)-PMF\left(\theta_{j}\right)$$

γ_*i*_ = λ^(2)^ – λ^(1)^

where PMF­(θ_*i*_) and PMF­(θ_*j*_) are substructured to avoid double-counting of the valence bending potential.

The fitted parameter set consisted of 21 coefficients. This functional form was adopted from ref. 44, where the full definitions of all the terms are described. The fitted function was used to construct the torsional potential term (eq 7) in the UNRES energy function.

7 $$\begin{array}{ll} U_{tor}\left(\gamma_{i},\theta_{i},\theta_{i+1}\right)= & \frac{1}{2}\sin\theta_{i}\sin\theta_{i+1}\{\left[\overset{2}{\underset{k=0}{\sum }}\overset{2}{\underset{l=0}{\sum }}\left(b_{X21}^{\left(k\right)}b_{Y11}^{\left(l\right)}+b_{X22}^{\left(k\right)}b_{Y12}^{\left(l\right)}\right){\left(\cos\theta_{i}\right)}^{k}{\left(\cos\theta_{i+1}\right)}^{l}\right]\cos\gamma_{i}\\ & +\left[\overset{2}{\underset{k=0}{\sum }}\overset{2}{\underset{l=0}{\sum }}\left(b_{X22}^{\left(k\right)}b_{Y11}^{\left(l\right)}+b_{X21}^{\left(k\right)}b_{Y12}^{\left(l\right)}\right){\left(\cos\theta_{i}\right)}^{k}{\left(\cos\theta_{i+1}\right)}^{l}\right]\sin\gamma_{i}\}\\ & -\frac{1}{4}{\left(\sin\theta_{i}\sin\theta_{i+1}\right)}^{2}\{\left[\overset{1}{\underset{k=0}{\sum }}\overset{1}{\underset{l=0}{\sum }}\left(c_{X11}^{\left(k\right)}d_{Y11}^{\left(l\right)}-c_{X12}^{\left(k\right)}d_{Y12}^{\left(l\right)}\right){\left(\cos\theta_{i}\right)}^{k}{\left(\cos\theta_{i+1}\right)}^{l}\right]\cos\left(2\gamma_{i}\right)\\ & +\left[\overset{1}{\underset{k=0}{\sum }}\overset{1}{\underset{l=0}{\sum }}\left(c_{X12}^{\left(k\right)}d_{Y11}^{\left(l\right)}+c_{X11}^{\left(k\right)}d_{Y12}^{\left(l\right)}\right){\left(\cos\theta_{i}\right)}^{k}{\left(\cos\theta_{i+1}\right)}^{l}\right]\sin\left(2\gamma_{i}\right)\} \end{array}$$

For the details and full derivation of the expansion, see ref. 45.

### 2.4 All-Atom and Coarse-Grained Simulations

Three sets of molecular dynamics simulations were used and performed in this study:

(i) An initial 500 ns long, all-atom simulation for 3K was used to parametrize the bond-stretching potential in the UNRES force field and a 500 ns long, all-atom simulation for PeptideIV was used to compare secondary structure reproduction of our parametrization work; (ii) test coarse-grained simulations with both 3K and EK; and (iii) additional all-atom simulations with both peptides for comparison with the coarse-grained results.

(i) We have used a 3K simulation from our earlier published work for parametrizing the bond-stretching term. The details of that simulation can be found in Kamal et al.^22^ Also, a prior all-atom simulation for PeptideIV was used for comparison; the details of that simulation can be found in Wacha et al.^40^

(ii) The test coarse-grained simulations were performed with 3K, EK, and PeptideIV β-peptides in water systems using the NEW-SP-CT force field available in UNRES with our modified parameters. All simulations were carried out in the canonical (NVT) ensemble, with the temperature maintained at 300 K using a Langevin thermostat. The simulation time varied between systems; the time step was 0.5 fs, and 4 parallel trajectories were done for each system. Following Liwo et al.,^47^ we adopted the commonly used scaling factor of 1000 to convert UNRES simulation time to experimental time.^48^ Scale friction was set to 0.02. Initial structures were energy-minimized prior to the production run.

The systems were the following: (1) 1 3K in water, box size was (150 Å × 150 Å × 150 Å), simulation time was 200000 MTU, corresponding to roughly 10 μs experimental simulation time.

(2) 1 EK in water, box size was (150 Å × 150 Å × 150 Å), simulation time was 200000 MTU, corresponding to roughly 10 μs experimental simulation time.

(3) 1 PeptideIV in water, box size was (150 Å × 150 Å × 150 Å), simulation time was 200000 MTU, corresponding to roughly 10 μs experimental simulation time.

(4) 64 molecules of 3K in water, box size was (300 Å × 300 Å × 300 Å), simulation time was 3000000 MTU, corresponding to roughly 150 μs simulation time.

(5) 64 molecules of EK in water, box size was (300 Å × 300 Å × 300 Å), simulation time was 500000 MTU, corresponding to roughly 23 μs simulation time.

(iii) All-atom molecular dynamics simulations, conducted for comparison with the monomer UNRES coarse-grained simulations, were performed using the CHARMM36 force field extended for β-peptides. Individual 3K and EK were simulated separately in explicit water with a physiological salt concentration, employing a cubic simulation box with an edge length of 4–5 nm. System equilibration was carried out using the V-rescale thermostat and a Berendsen barostat. For each peptide, four independent trajectories were generated, each having a 1000 ns production run using a 2 fs time step. Electrostatics were handled by the Particle Mesh Ewald (PME) method; the LINCS algorithm was used to constrain bonds between hydrogens and their corresponding heavy atoms.

The simulations were evaluated using in-house Fortran and Python scripts and MDAnalysis.^49,50^ The radius of gyration (*R*_*g*_), end-to-end distance, LYS-LYS distances, and Cβ-Cβ distances (termed Cα in our model) were compared to values found in all-atom simulations of 3K. The same attributes except for LYS-LYS distances were compared for EK with the corresponding all-atom simulations. The results from 4 trajectories were averaged. The results for EK simulations can be found in Supporting Information; here we present only the results for 3K.

Images illustrating simulation results were produced using PyMOL.^51^

## 3 Results

### 3.1 Bond-Stretching Potential (*U*_*bond*_)

The distribution of distances between neighboring β^3^-carbon atoms (Cβ) was recorded at 1 ns intervals across the trajectory (Figure 5).

**Figure 5:**
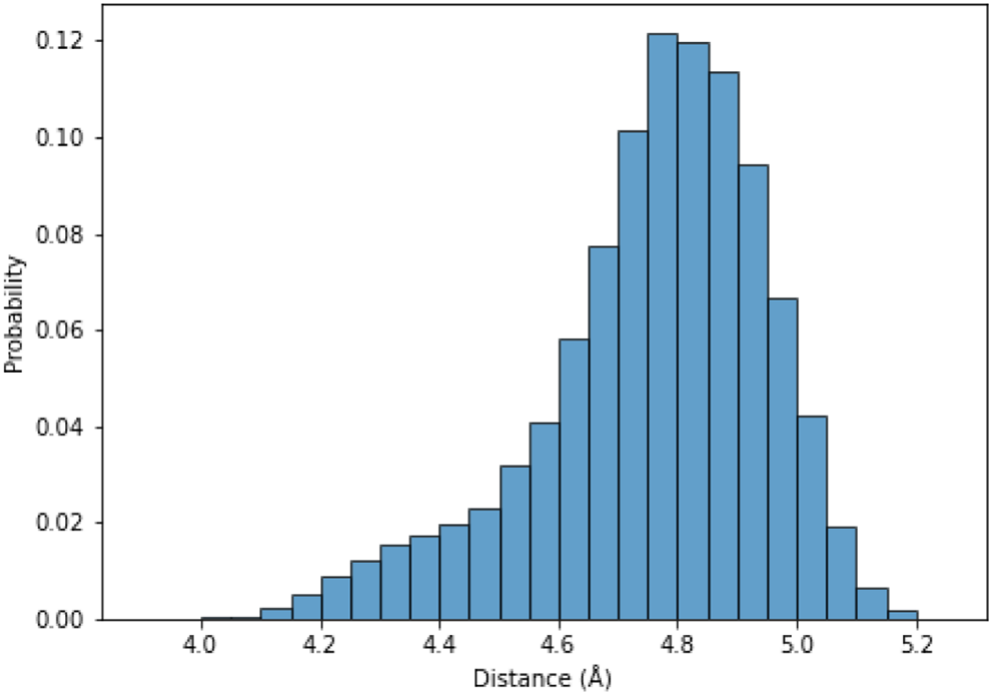
Probability distribution of distances between all neighboring β-carbon pairs in 3K. The *y*-axis shows the normalized probability, and the *x*-axis shows the inter-β-carbon distance (Å). Histograms were computed by using a bin width of 0.05 Å.

From the normalized histogram values, the potential of mean force (PMF) was calculated by using the standard Boltzmann inversion:

The minimum distance *x*_0_ value was taken from the minimum value of the PMF curve, which was 4.8 Å. The *k* (eq 8) constant was calculated based on two adjacent bin points of the minimum using the following formula, where *i* = 0.05:

8 $$k=\frac{2\left(PMF\left(x_{0}+i\right)-PMF\left(x_{0}-i\right)\right)}{{\left((x_{0}+i)-(x_{0}-i)\right)}^{2}}=\frac{2\left(1.35-1.26\right)}{0.1^{2}}=18$$

The resulting PMF values are reported in units of kcal/mol. The fitted curve was shifted by the minimum of the PMF curve for comparison (Figure 6). While the harmonic potential qualitatively captures the curvature of the PMF near its minimum, deviations increase at longer and shorter distances. The RMSD between the harmonic fit and the PMF is 1.004 kcal/mol.

**Figure 6:**
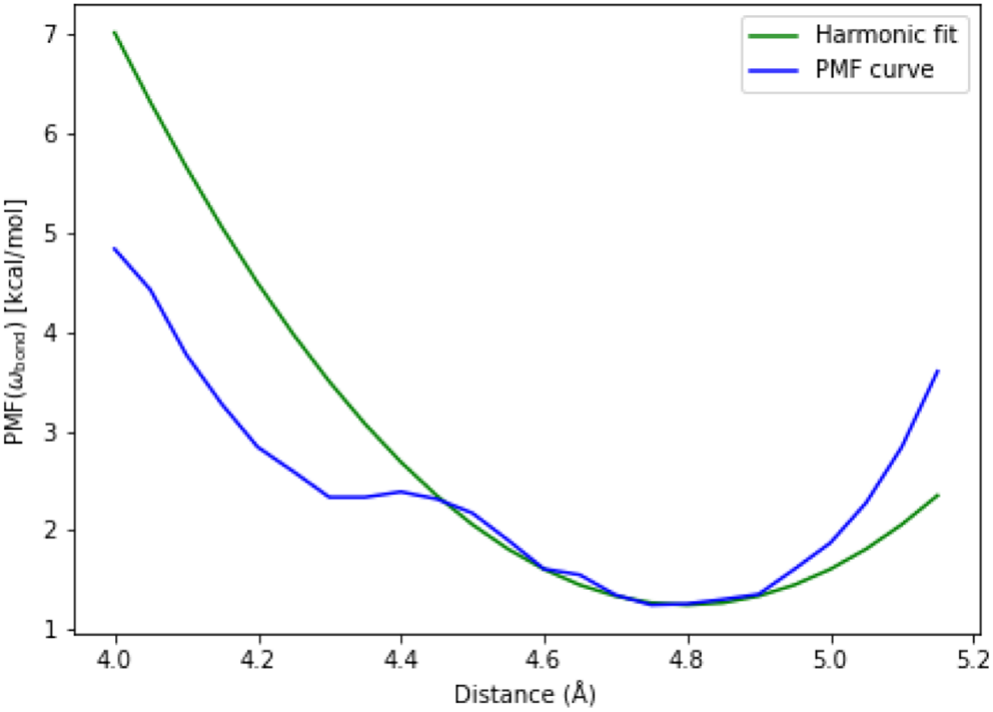
Potential of mean force (PMF) profile for the ω-bond length in kcal · mol^–1^, with the *x*-axis showing the corresponding bond distance in Å. Both the PMF curve obtained from the simulation data and the fitted harmonic potential are displayed.

### 3.2 Virtual Bond Angle Bending Potential (*U_b_*)

The potential of mean force (PMF) associated with the backbone virtual bond angle θ was derived from quantum chemical energy scans, as described above. To isolate the dependence on θ, energies computed over a three-dimensional grid of (λ^(1)^,λ^(2)^,θ) were processed by using Boltzmann reweighting. For each fixed value of θ, a PMF was computed by summing the Boltzmann-weighted energy contributions over all λ^(1)^ and λ^(2)^ combinations, according to 4. The calculated energy values were first shifted relative to the minimum energy in the dataset and converted from Hartrees to kcal/mol using a factor of 627.51; see eq 9 for obtaining this conversion factor.

9 $$\begin{array}{ll} 1\mathrm{Eh} & =\frac{4.359744722\times 10^{-18}J}{4184{J\mathrm{kcal}}^{-1}}\times 6.02214076\times 10^{23}{\mathrm{mol}}^{-1}\\ & =627.51{\mathrm{kcal}\mathrm{mol}}^{-1} \end{array}$$

The summation over λ angles was performed within 5° bins for θ, resulting in a 1D PMF profile. This PMF was used as a reference for fitting the bond angle potential. The fitted angular potential was constructed by using eq 5. Figure 7 compares the fitted curve with the PMF profile obtained from the quantum chemical data.

**Figure 7:**
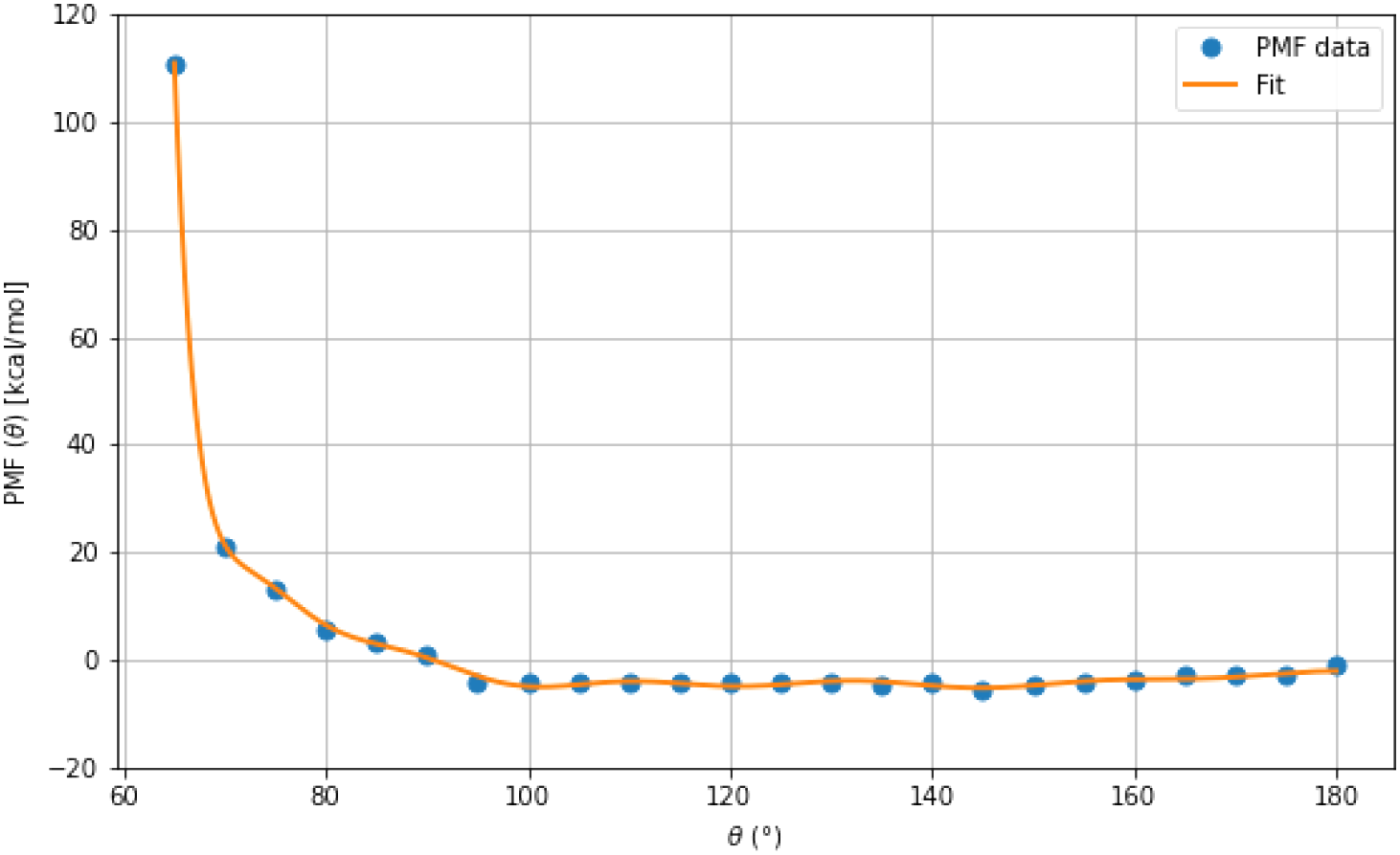
Potential of mean force (PMF) profile for the θ angle, together with the fitted angular potential. The *y*-axis shows the PMF­(θ) in kcal · mol^–1^, and the *x*-axis shows the corresponding θ angle in degrees. Both the PMF data obtained from the θ scan calculations and the fitted function are shown.

The fitted virtual-valence angle bending potential accurately reproduced the PMF profile, yielding a root-mean-square deviation (RMSD) of 0.496 kcal/mol, indicating excellent agreement with the reference PMF. The fitted coefficients are shown in Table S2.

### 3.3 Torsional Potential (*U*_*tor*_)

Energy grid was computed in three stages: one with fixed λ^(1)^ = 0 and varied λ^(2)^ as a scan and θ was minimized; another with fixed λ^(2)^ with the value from the previous scan and varied λ^(1)^ and θ was minimized; and finally, a scan in θ with fixed λ^(1)^ and λ^(2)^ was performed. Energies were first shifted relative to the global minimum and converted from Hartrees to kcal/mol using a factor of 627.51. The PMF was computed via Boltzmann inversion of the total energy contributions from both grids (eq 6). The summation was performed over all grid combinations of λ^(1)^, λ^(2)^, and the resulting torsional angle γ_*k*_ = λ^(2)^ – λ^(1)^. The final PMF surface was output as a function of θ_*i*_, θ_*j*_, and γ_*k*_, binned at 5° increments in θ and 15° in γ.

The fitted torsion potential accurately reproduced the PMF surface in most regions, as shown in Figure 8. It should be noted the fit did not reproduce the surface reliably in high-energy regions. Since there was a discrepancy in the fit correlation between different parts of the surface, we calculated the mean absolute error (MAE) to quantify the overall error of the fit. The MAE yielded 3.56 kcal/mol, which indicates that, on average, the fitted potential deviates moderately from the reference PMF across the grid; however, the most discrepancies arise from high-energy regions, which are of less interest. The fitted coefficients are shown in Table S3.

**Figure 8:**
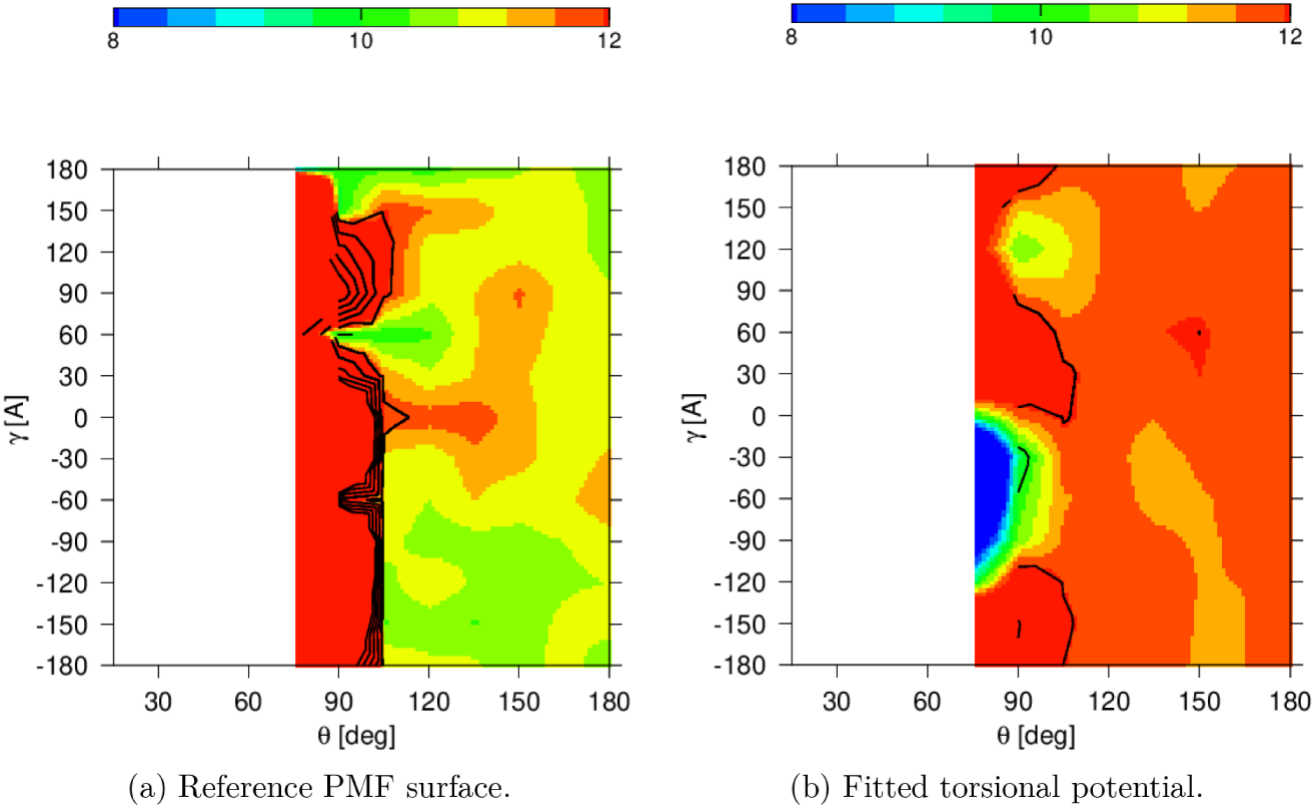
Comparison of the torsional potential energy surfaces for θ and γ: (a) Reference PMF surface. (b) Fitted torsional potential. Both panels show 2D energy landscapes as functions of θ and γ, where the color scale corresponds to the energy in kcal · mol^–1^.

### 3.4 Monomer 3K and EK Simulations

Here we compare mainly the monomer 3K UNRES test simulations with all-atom equivalent simulations. In the Supporting Information, you can find the figures for the same comparisons between the EK all-atom and coarse-grained simulations. Note, the simulation time of the all-atom systems was 1000 ns, while the duration of the coarse-grained simulation was around 10 μs. The Cβ-Cβ distance distributions for all-atom simulation have already been presented for 3K by Figure 5, and here we show the comparison between the coarse-grained UNRES simulations and the all-atom simulations (Figure 9). In Kamal et al., NMR results indicate random coil conformation of 3K;^22^ in our simulation the peptide shows no definite secondary structure, which shows correspondence with the NMR experiment. Next, we compared the length, angle, and dihedral angle distributions of the Cβ atoms for 3K.

**Figure 9:**
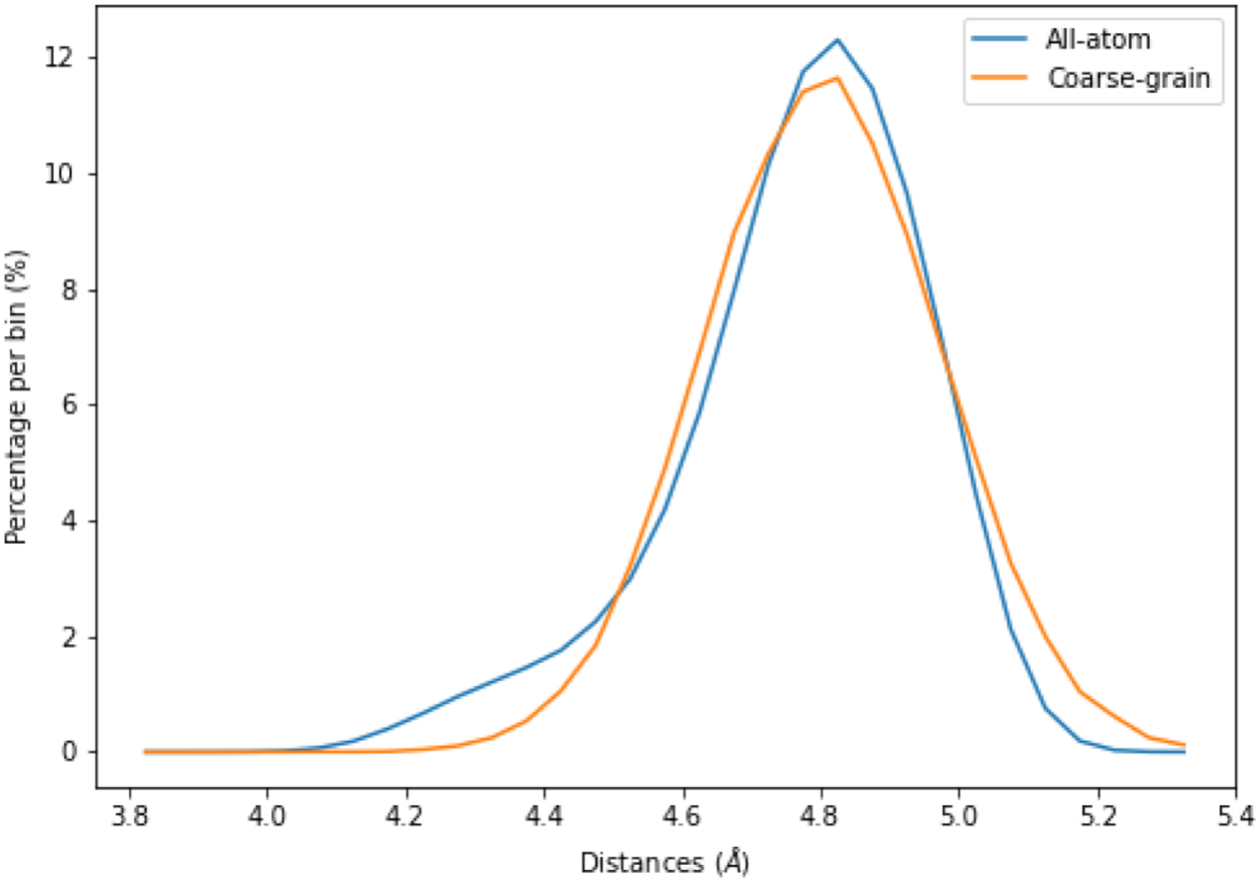
Distance distribution between neighboring β-carbon atoms in 3K from all-atom and coarse-grained simulations. The *y*-axis shows the normalized probability density, and the *x*-axis shows the inter-β-carbon distance in Å. Curves corresponding to the all-atom (AA) and coarse-grained (CG) simulations are shown.

The all-atom and coarse-grained histograms of Cβ-Cβ distances had an RMSD of 0.151 between the normalized distributions, confirming that the fitted bond potential reproduces the reference ensemble with a high accuracy. In Figure S1, the same comparison curves between the distances can be seen for EK.

The distribution of all 3 neighboring Cβ atoms’ angles showed significant differences between the AA and CG simulations (Figure 10). The most frequent angles were around 100°, and the CG simulation had 150° overrepresented. The angle distributions are practically identical for EK as for 3K (Figure S2). The largest differences in distributions are observed for the torsional angles (Figure 11). In UNRES, conformational changes happen faster due to coarse-graining; therefore, a more flexible chain is observed, which leads to a flatter distribution of torsion angles. However, it should be noted that in the all-atom force field, the barrier for the rotation of the Cβ-Cβ-Cβ-Cβ angle is very small (≈0.5 kcal/mol), in contrast to α-peptides, for which the corresponding barrier is approximately 3 kcal/mol. This low torsional barrier reflects the greater conformational freedom of β-peptides^52^ and is further supported by their smaller effective spring constants compared to α-peptides (18 kcal/mol/Å^2^ vs 41.7 kcal/mol/Å^2^). Because the torsional energy barriers are extremely low, accurately reproducing the corresponding energy profiles required sub-0.5 kcal/mol precision. In this regime, the coarse-grained representation, whose fitting errors exceed this energy scale (RMSE > 3 kcal/mol), cannot fully capture subtle variations in torsional preferences and primarily reflects the absence of significant torsional barriers. Furthermore, in Supporting Information it can be seen that in the case of EK, both all-atom and coarse-grained simulations produce a flat distribution of torsional angles (Figure S3).

**Figure 10:**
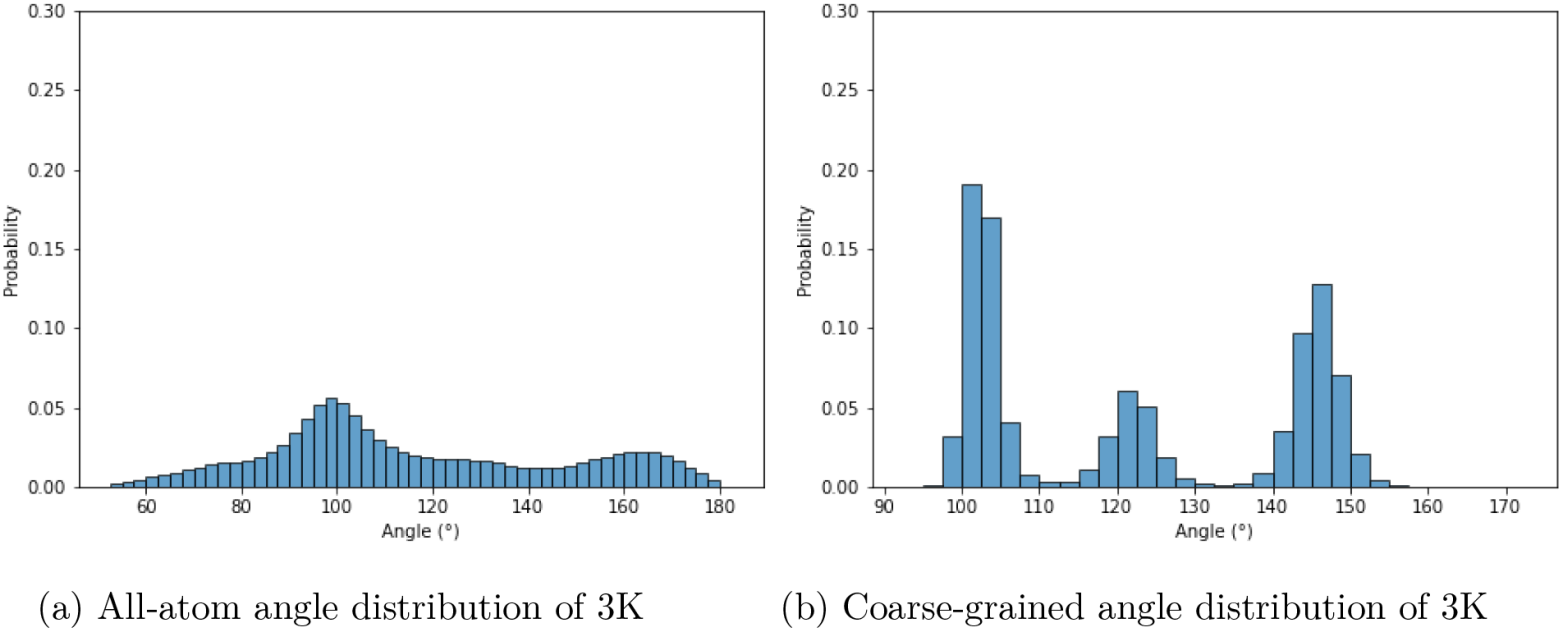
Comparison of the angle distributions defined by each triad of neighboring Cβ atoms in 3K. Panel (a) shows data for all-atom simulations, and panel (b) shows data for coarse-grained simulations. The *y*-axis shows normalized probability density, and the *x*-axis shows the angles measured between the carbon atoms in degrees.

**Figure 11:**
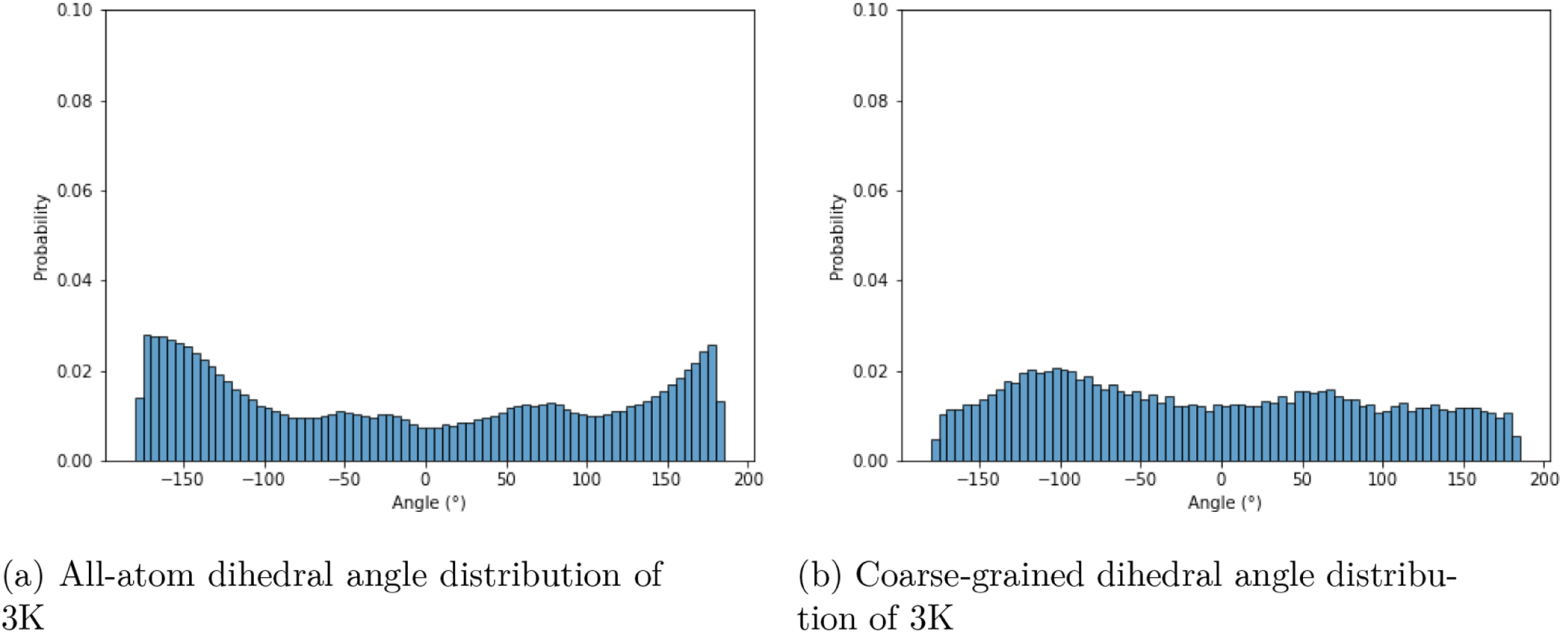
Comparison of the dihedral angle distributions defined by each quartet of neighboring Cβ atoms in 3K. Panel (a) shows data for all-atom simulations, and panel (b) shows data for coarse-grained simulations. The *y*-axis shows normalized probability density, and the *x*-axis shows the dihedral angles measured between the carbon atoms in degrees.

The radius of gyration (Rg) was computed for both all-atom and coarse-grained simulations to assess the global compactness of the peptides. The average Rg value obtained from the all-atom simulation was 7.470 ± 0.684 Å, while the coarse-grained simulation yielded an average of 7.418 ± 0.533 Å (Figure 12). The Rg of EK in both all-atom and coarse-grained simulations was comparable as well (Figure S4). This result further confirms the correct parametrization of our coarse-grained force field.

**Figure 12:**
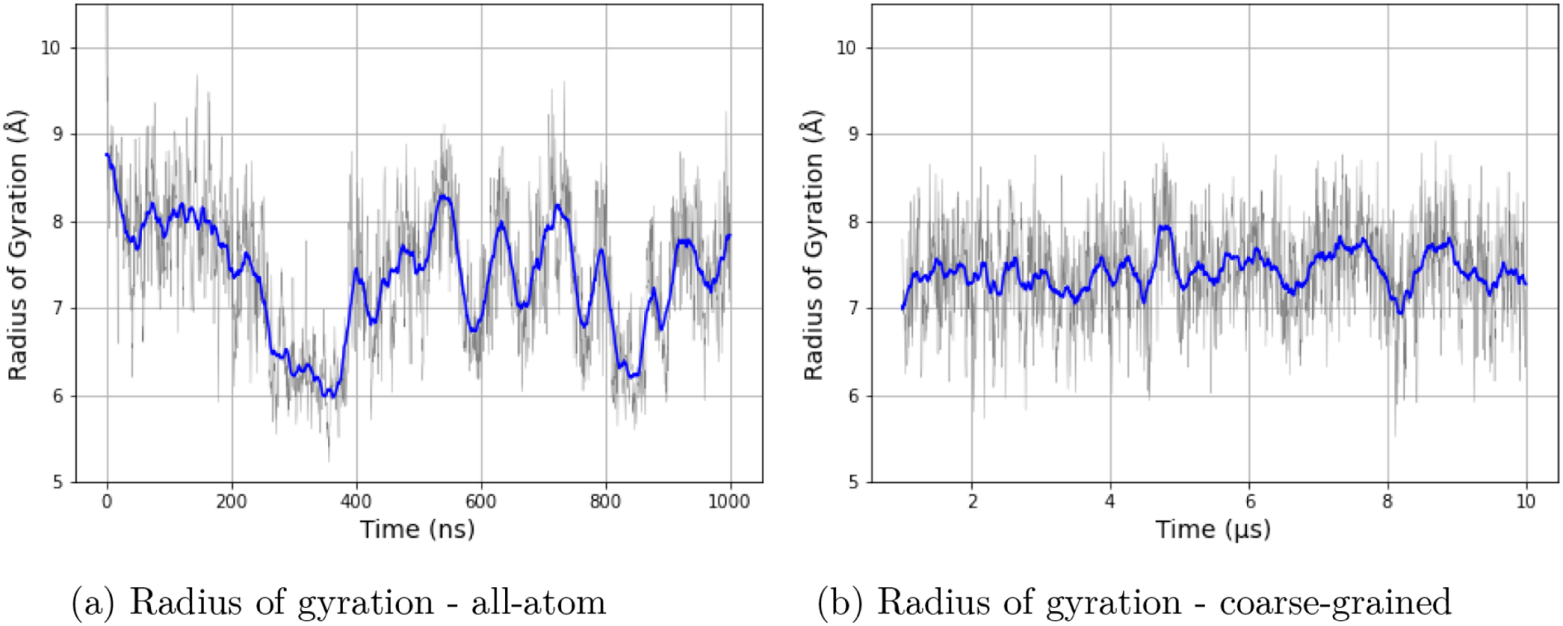
Radius of gyration values of 3K in all-atom and coarse-grained simulations. Panel (a) shows data for all-atom simulations, and panel (b) shows data for coarse-grained simulations. The *y*-axis shows the radius of gyration values in Å, and the *x*-axis shows the time of the simulations.

To evaluate the overall extension of the peptide conformations, the end-to-end distance was calculated as the distance between the backbone carbon atoms of the first and last residues. Specifically, the positions of the Cβ atoms (in the coarse-grained model) and the corresponding backbone carbon atoms (in the all-atom model) were used to define the termini. The average end-to-end distance was 17.091 ± 2.953 Å in the all-atom simulations and 18.175 ± 2.893 Å in the coarse-grained simulations. The average value and distribution of end-to-end distances show remarkable agreement (Figure 13), which was the case for EK as well (Figure S5).

**Figure 13:**
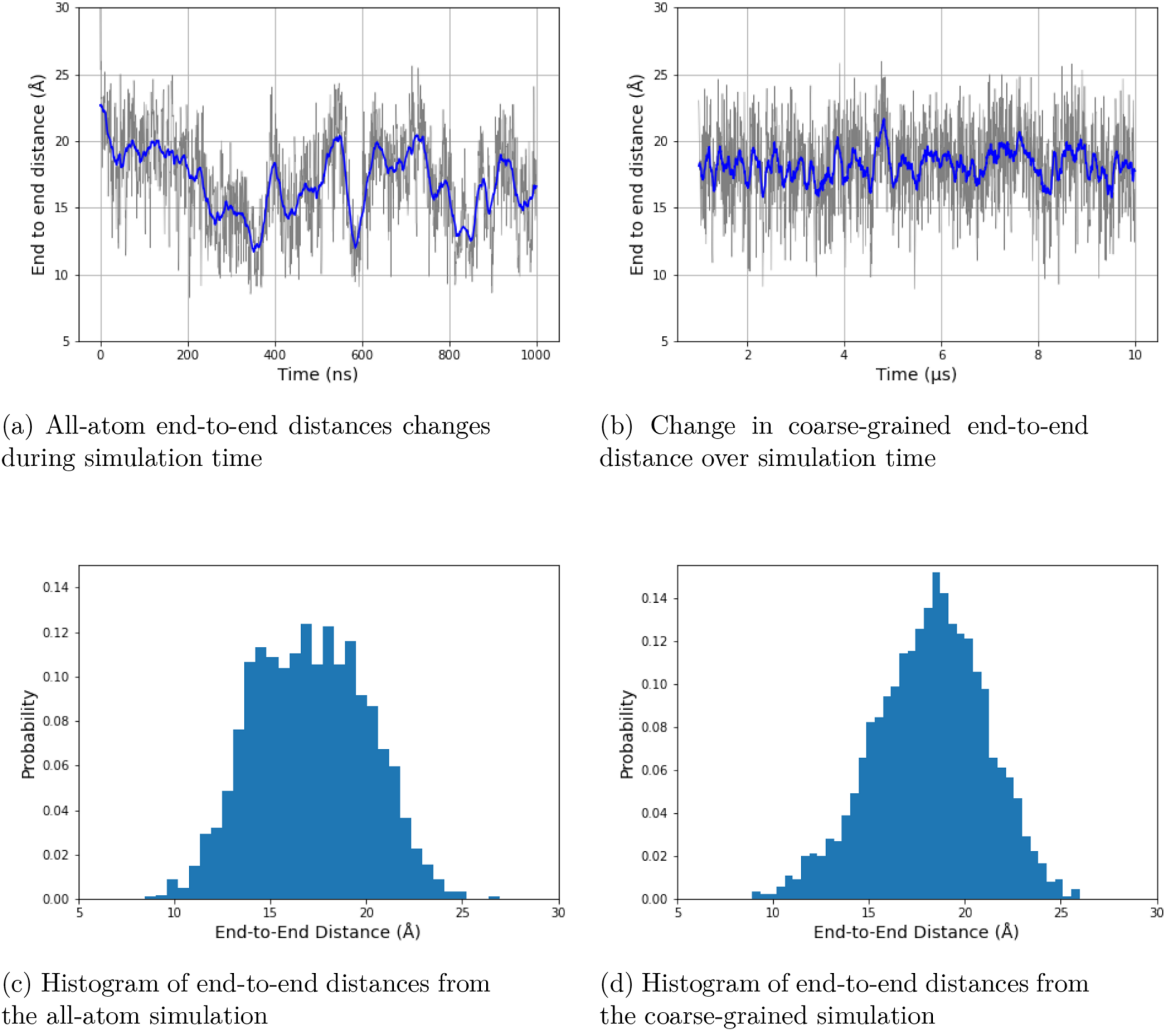
End-to-end distances comparison for 3K in all-atom and coarse-grained simulations. Panel (a) shows data for all-atom simulations, and panel (b) shows data for coarse-grained simulations. The end-to-end distance was defined as the distance between the first and last β-carbon atoms of the peptide. The *y*-axis shows the end-to-end distance values in Å, and the *x*-axis shows the time of the simulations for panels (a) and (b). The *y*-axis shows the normalized probability density, and the *x*-axis shows the end-to-end distance distribution values in Å for panels (c) and (d).

An important feature of 3K peptide is the distance between its lysine residues; thus, we analyzed the distances between three lysine side-chain pairs along the peptide sequence. The distances were measured between the third and fifth, the fifth and seventh, and the third and seventh lysine residues’ center of mass in 3K for all-atom and coarse-grained simulations (Figure 14). The average lysine–lysine distances were computed for each pair, and the results showed moderate deviations between the two models (Table 1). This comparison provides insight into how well the coarse-grained force field captures the spatial arrangement and flexibility of charged side chains that are critical for the peptide’s structural behavior. EK does not have important side-chain distance properties, so there was no similar analysis done for the EK simulations.

**Table 1:** Lysine–Lysine Distances in Å

Simulation residue	Average ± std
AA 3LYS-5LYS	8.827 ± 0.833
CG 3LYS-5LYS	8.638 ± 0.305
AA 5LYS-7LYS	8.770 ± 0.761
CG 5LYS-7LYS	8.923 ± 0.637
AA 3LYS-7LYS	13.276 ± 1.694
CG 3LYS-7LYS	14.464 ± 1.417

**Figure 14:**
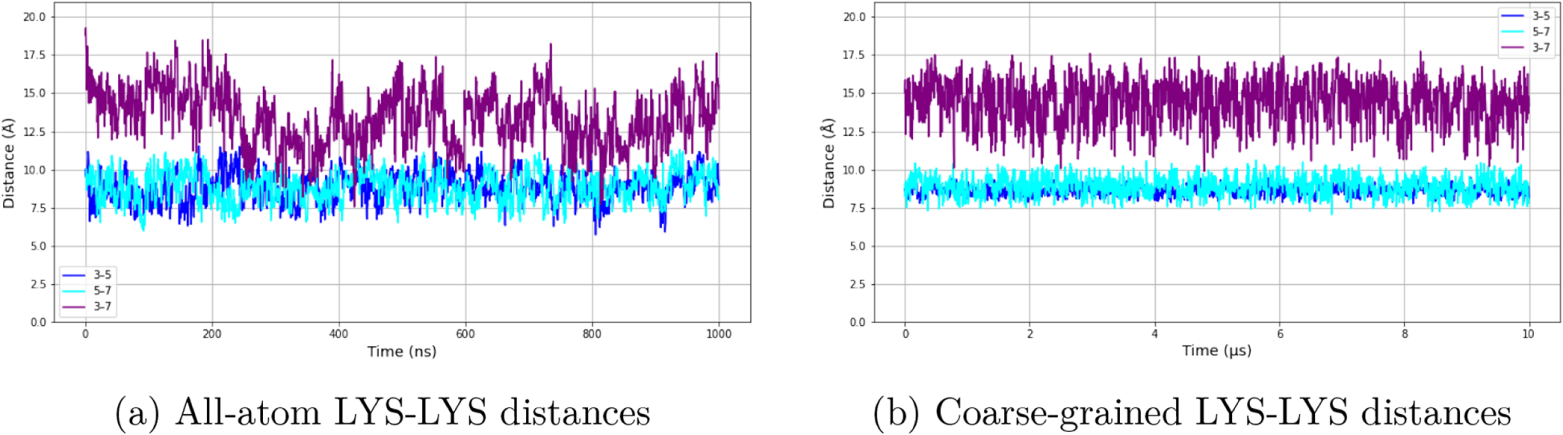
Comparison of LYS-LYS distances in 3K from all-atom and coarse-grained simulations. Panel (a) shows data for all-atom simulations, and panel (b) panel shows data for coarse-grained simulations. The plots show the center-of-mass distances between three lysine residue pairs: LYS3-LYS5, LYS3-LYS7, and LYS5-LYS7. The *y*-axis shows the center-of-mass distances between the selected residues in Å, and the *x*-axis shows the time of the simulations.

### 3.5 Secondary Structure Study

In order to assess the reproducibility of the secondary structure, we investigated PeptideIV, which is experimentally reported to adopt a helical structure in water. In our simulations, the increased backbone flexibility prevented the formation of a fully stable helical structure over the trajectories. Nevertheless, cluster analysis revealed that the most prevalent cluster corresponds to a conformation that closely resembles a helical structure. A comparison with the most prevalent cluster obtained from all-atom simulation shows very good agreement with global structural properties. The coarse-grained cluster exhibits a radius of gyration of 6.36 and an end-to-end distance of 16.02 Å, while the corresponding all-atom cluster has values of 6.83 and 16.08 Å, respectively. Moreover, in the UNRES simulations, 62% of the trajectory frames were assigned to this dominant cluster, indicating that this helix-like conformation represents the prevailing structural motif sampled by the system. Figure 15 shows the helical structure obtained with UNRES and compared to the helical structure from the all-atom simulation of PeptideIV.

**Figure 15:**
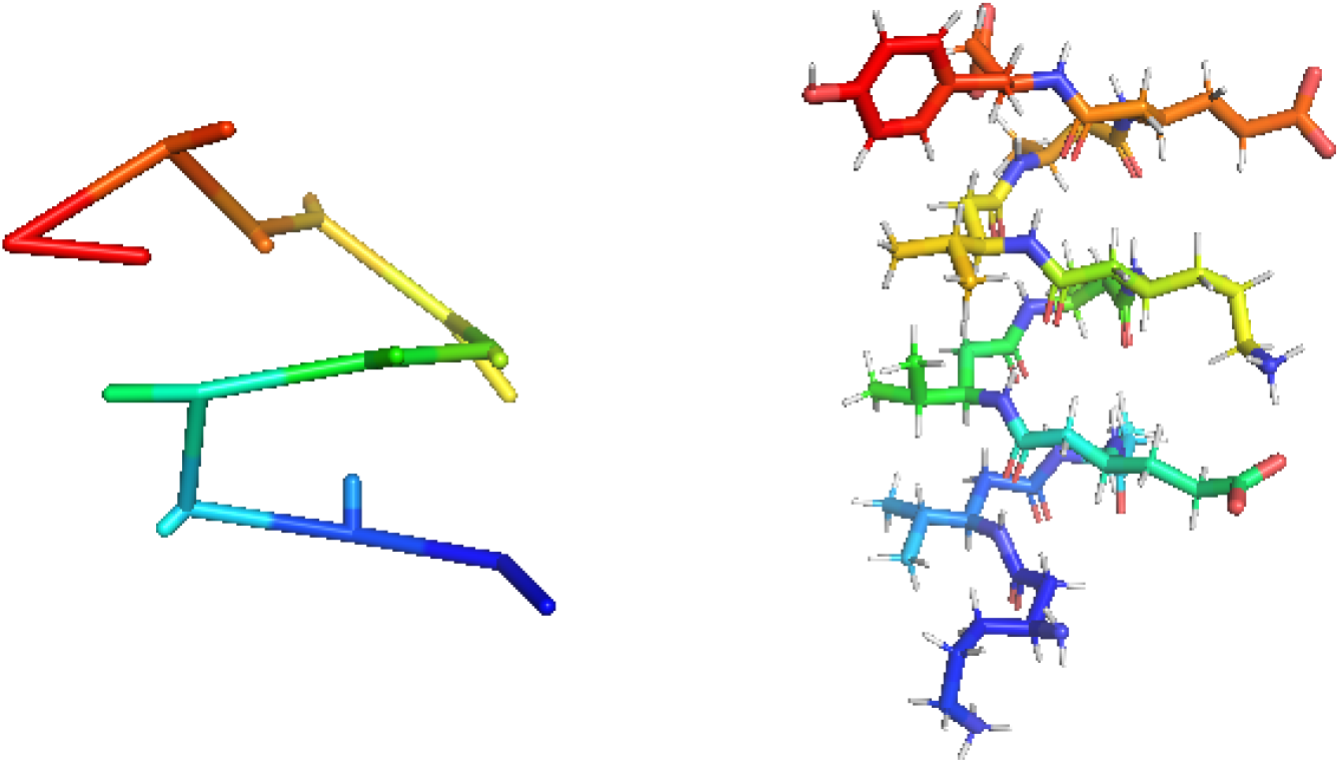
Comparison of the dominant helical conformations of PeptideIV obtained from UNRES (left) and all-atom (right) simulations. Structures correspond to the centroids of the most populated clusters identified by the cluster analysis.

### 3.6 Self-Assembly Study with 64 Peptide-Containing Systems

To allow sufficient time for the formation of larger assemblies, this simulation was conducted for a substantially longer duration compared to the monomer simulations, reaching a total simulation time of almost 0.15 ms in the case of 3K, and around 25 μs in the case of EK. The shorter time for EK was used as we found that EK associates extremely fast. However, even with the longer trajectory, only multiple smaller assemblies were observed in the case of 3K. Cluster analysis revealed that only small aggregates can be found, as can be seen in the distributions of the aggregate size for the 5 most prevalent clusters from the simulation (Figure 16) In one cluster, the highest aggregate formed consists of 6 3K molecules, but generally many smaller aggregates formed (3–5); however, no larger aggregates were detected throughout the extended simulation. This observation is consistent with previous experimental findings indicating that this peptide does not form large assemblies in pure solution^53^ (Figure 17a). As already mentioned before, for EK, one big aggregate formed, including all 64 peptides in the simulations (Figure 17b) for 8 out of the top 10 dominant clusters (in two cases, one monomer dissociated). To quantify the compactness of the peptide aggregates, we computed the radius of gyration (Rg) and the corresponding maximum radius of gyration (Rg_*max*_) for the 8 most probable clusters. The Rg_*max*_ is the maximum possible radius of gyration for a cluster. The probability-weighted average value of Rg was 21.72 ± 1.21 Å, and the probability-weighted average of Rg_*max*_ was 39.04 ± 3.83 Å. This indicates that the peptide assembly is considerably smaller than the theoretical maximally extended conformation. It is noteworthy that the assemblies adopt a double array architecture, in which two layers of EK peptides align in an ordered manner.^22,53^ In this configuration, the hydrophobic leucine residues predominantly orient toward the interior of the double array, while the hydrophilic lysine and glutamate residues face outward. In the simulations involving 64 EK peptides, multiple smaller double array units formed and were associated with each other in a less ordered, heterogeneous manner (Figure 18). Overall, our 64-mer simulation shows that the EK peptide robustly self-assembles into higher-order structures, in line with experimental evidence that this peptide forms stable aggregates in aqueous environments.^54^ Thus, our model correctly reproduced the mesoscale phenomenon of self-assembly in the case of a β^3^-peptide, which is capable of self-assembly in water, proving that our parametrization work is suitable for predicting the self-assembly of β^3^-peptides.

**Figure 16:**
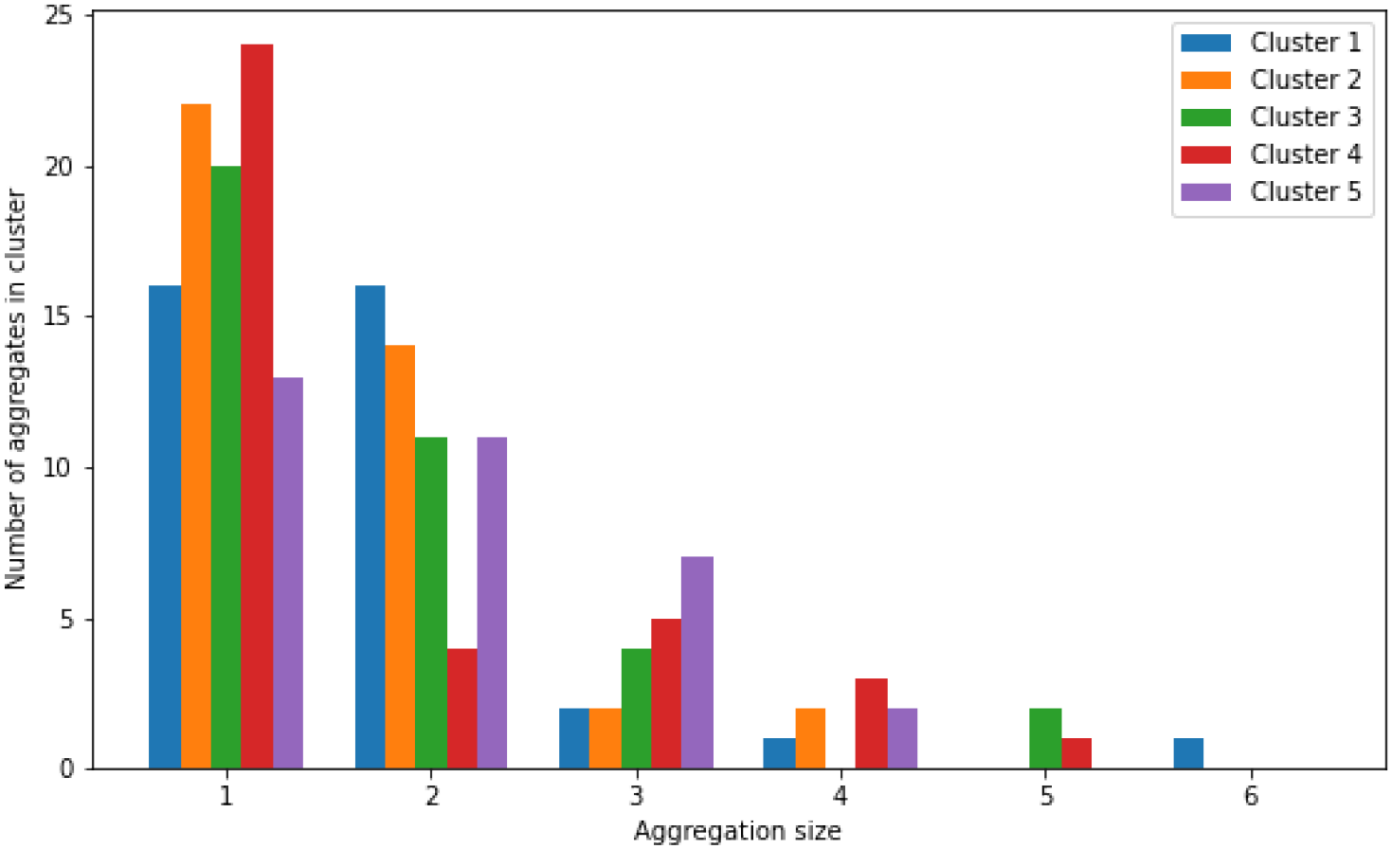
Aggregate size distribution across the 5 most prevalent clusters from the 64 3K simulations. One shows the number of monomers found in the clusters, 2 shows the number of dimers found in the clusters, 3 shows the number of trimers found in the clusters, 4 shows the number of tetramers found in the clusters, 5 shows the number of pentamers found in the clusters, and 6 shows the number of hexamers found in the clusters.

**Figure 17:**
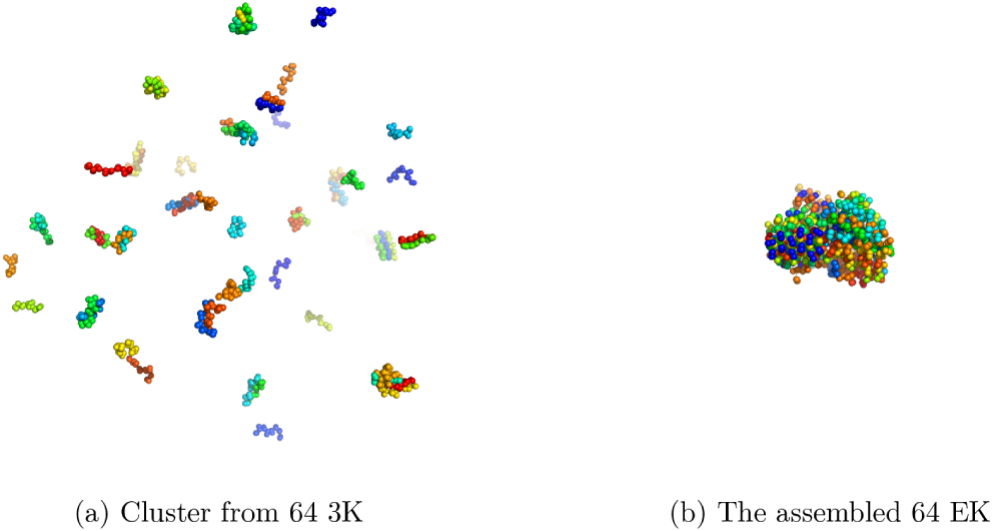
Most prevalent clusters from the 64-mer simulations. Panel (a) shows the cluster for 3K simulations, and panel (b) shows the cluster for EK simulations.

**Figure 18:**
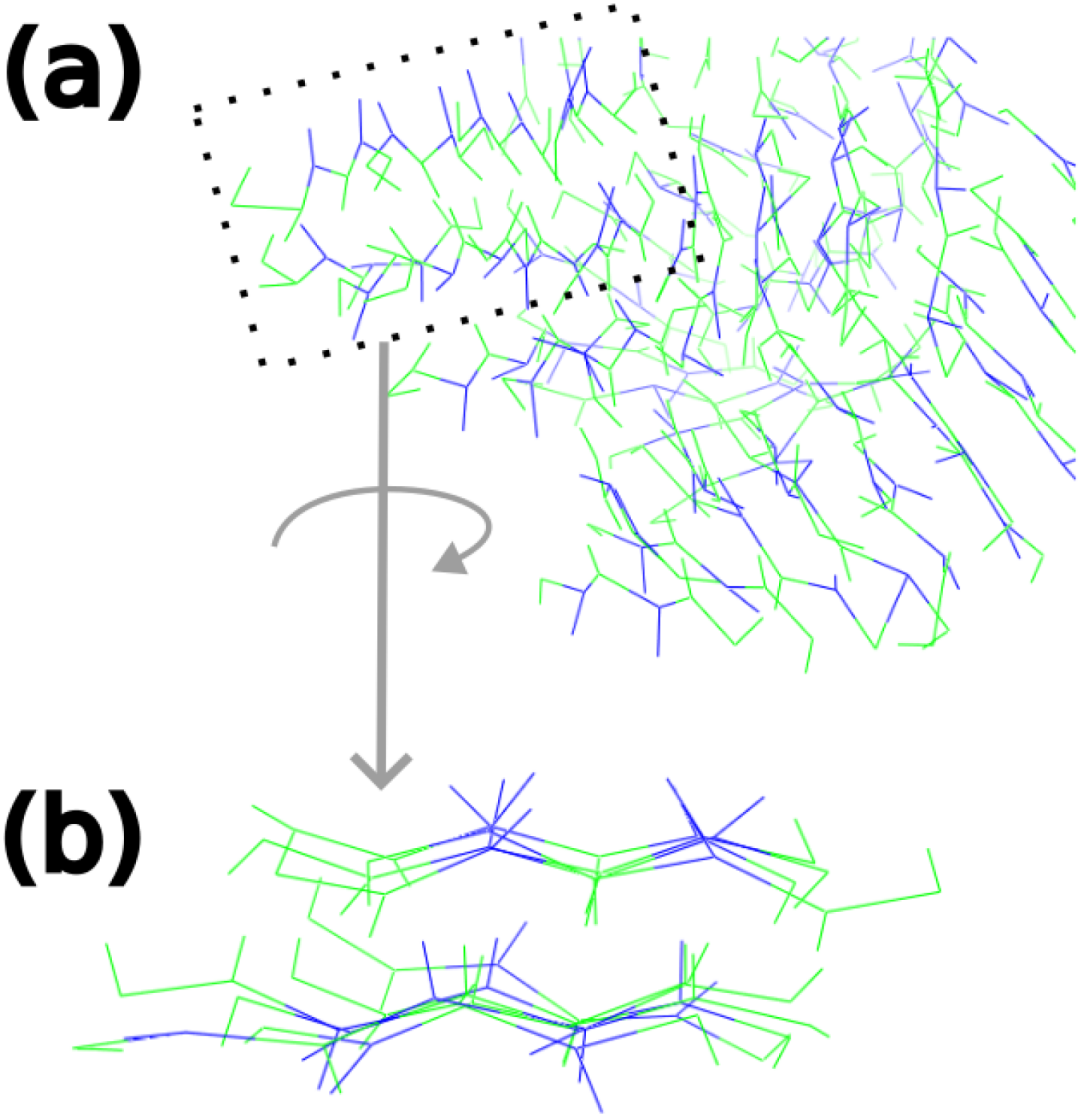
Representative double-array structure from the most prevalent cluster in the 64 EK simulations. Leucine residues are colored green, while lysine and glutamate residues are depicted in blue. In panel (a), a black rectangle highlights a single smaller double-array subunit within the larger assembly, which is magnified and rotated in panel (b).

## 4 Conclusion

In this study, we extended the UNRES force field to enable the simulation of β^3^-peptides. Three local energy terms of the UNRES coarse-grained force field were modified. The potential energy surface (PES) of a terminally blocked β^3^-amino acid residue was computed with the GFN2-xTB quantum chemical method by computation on the grid by scanning virtual angle and virtual torsional angle coordinates. Based on the resulting energy landscape, the corresponding local potentials in UNRES were adjusted in order to reflect the conformational preferences of β^3^-peptides. To evaluate the performance of the modified force field, we carried out molecular dynamics simulations of β^3^-peptides in aqueous solution and compared the results to available all-atom simulation data for each peptide. Distances, angles, and torsional angles between Cβ atoms were analyzed. Distances were reproduced with high accuracy, while angles showed moderate deviations, and torsion angle distributions differed, as expected due to coarse-graining. We further assessed global structural properties, including the radius of gyration and end-to-end distance. These properties closely matched those observed in all-atom simulations. Secondary structure reproduction was evaluated using an appropriate peptide suitable for this task; although a fully stable helix was not formed, cluster analysis revealed a dominant helix-like conformation. In addition, the self-assembly behavior of peptides was tested by simulating systems with 64 peptide molecules using the modified UNRES model. The 64-mer systems formed a big aggregate in the case of the peptide, which is capable of self-assembly; meanwhile, the other did not form big aggregates, which were in correspondence with experimental results for these β-peptides. Overall, the extended UNRES model demonstrated reliable performance in reproducing key structural and assembly characteristics of β^3^-peptides, showed moderate reliability in predicting secondary structure, and the modified parametrization provides a practical balance between computational efficiency and structural accuracy for large-scale coarse-grained simulations with UNRES.

## Supplementary Material



## Data Availability

The source code, binary, parameter files, example input, input generation protocol for β-peptides, and execution scripts are available at the UNRES website: https://unres.pl/.

## Notes

Weight factors for each UNRES energy equation terms (Table S1); fitted coefficients *a_n_* for the valence angle potential (Table S2); fitted coefficients (Table S3); distance distribution between neighbouring β-carbon atoms in EK from all-atom and coarse-grained simulations (Figure S1); comparison of the angle distributions defined by each triad neighbouring Cβ atoms in EK (Figure S2); comparison of the dihedral angle distributions defined by each quartet of neighbouring Cβ atoms in EK (Figure S3); radius of gyration values of EK in all-atom and coarse-grained simulations (Figure S4); end-to-end distances comparison for EK in all-atom and coarse-grained simulations (Figure S6) (PDF)
