# Supplementary material for: Parametrization of β3‑Peptides for Coarse-Grained Molecular Dynamics Simulations
Source: J Chem Inf Model. 2026 Mar 20;66(7):3955–68. doi: 10.1021/acs.jcim.5c03108 (PMC13080962; doi:10.1021/acs.jcim.5c03108)
Supplement: Supplementary file 1 [file ci5c03108_si_001.pdf]

## Supporting Information

# Parametrization of $\beta^3$-peptides for coarse-grained molecular dynamics simulations

O. Pavela[1,3], A. Wacha [1], T. Beke-Somfai*[1], A. Sieradzan*[2]

[1] Institute of Materials and Environmental Chemistry, HUN-REN Research Centre for Natural Sciences, Magyar tudósok körútja 2, H-1117 Budapest, Hungary

[2] Faculty of Chemistry, Laboratory of Molecular Modeling, University of Gdańsk, ul. Wita Stwosza 63, 80-380 Gdańsk, Poland

[3] Hevesy György Ph.D. School of Chemistry, Eötvös Loránd University, Budapest H-1117, Hungary

# 1 Tables

Table S1: Table S1: Weight factors for each UNRES energy equation (eq. 1) terms.

| term factor | value |
|---|---|
| $\omega_{SC}$ | 0.81230 |
| $\omega_{SCp}$ | 1.2042 |
| $\omega_{pp}^{VDW}$ | 0.33393 |
| $\omega_{pp}^{el}$ | 0.87690 |
| $\omega_{bond}$ | 1.0000 |
| $\omega_{ssbond}$ | 1.9773 |
| $\omega_{rot}$ | 0.063657 |
| $\omega_{tor}$ | 1.4154 |
| $\omega_{corr}^{(3)}$ | 0.036638 |
| $\omega_{turn}^{(3)}$ | 1.4511 |

Table S2: Table S2: Fitted coefficients $a_n$ for the valence angle potential $U_{bX}(\theta) = \sum a_n \cos(n\theta)$.

| $n$ | $a_n$ (kcal/mol) |
|---|---|
| 0 | 202444 |
| 1 | 387901 |
| 2 | 340385 |
| 3 | 273433 |
| 4 | 200061 |
| 5 | 132551 |
| 6 | 78819 |
| 7 | 41518.7 |
| 8 | 19004.1 |
| 9 | 7334.55 |
| 10 | 2268.46 |
| 11 | 509.136 |
| 12 | 63.8712 |

Table S3: Table S3: Fitted coefficients for equation 7

| coeff | value (kcal/mol) |
|---|---|
| e0 | 11.68705 |
| b1m(1,1) | 6.55666 |
| b1m(1,2) | 18.77555 |
| b1m(2,1) | 42.60326 |
| b1m(2,2) | 71.65283 |
| b1m(3,1) | 57.76376 |
| b1m(3,2) | 67.58073 |
| b2m(1,1) | 0.04421 |
| b2m(1,2) | -0.05351 |
| b2m(2,1) | 0.46195 |
| b2m(2,2) | -0.38883 |
| b2m(3,1) | 0.49912 |
| b2m(3,2) | -0.31913 |
| cm(1,1) | -0.40665 |
| cm(1,2) | 2.42886 |
| cm(2,1) | -0.87062 |
| cm(2,2) | 5.34361 |
| dm(1,1) | -2.88635 |
| dm(1,2) | 1.67487 |
| dm(2,1) | -6.60467 |
| dm(2,2) | 1.51498 |

# 2 Additional Figures

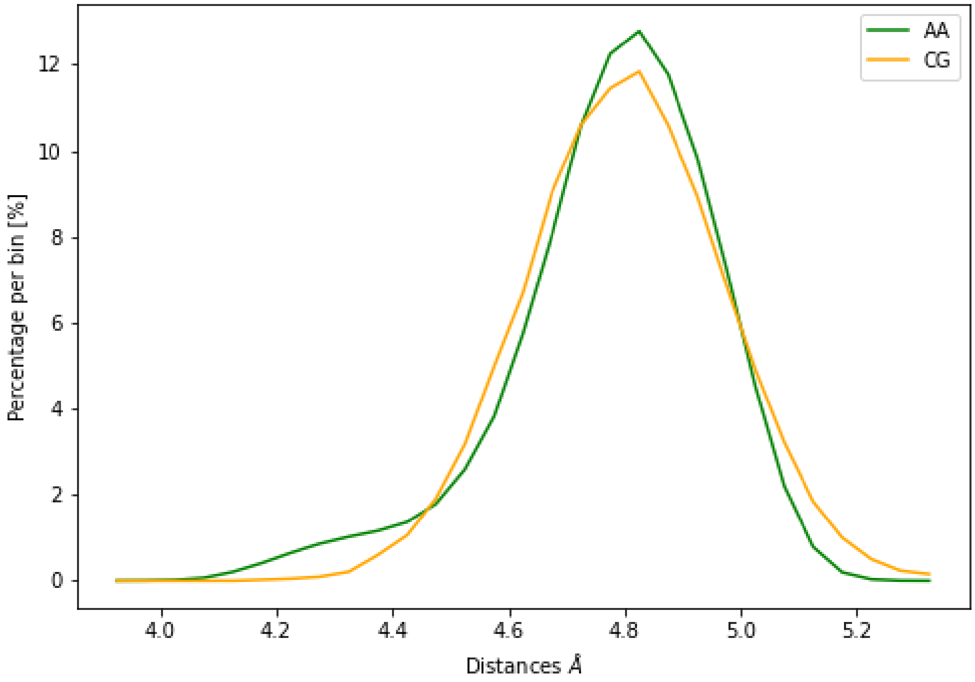


Figure S1: Distance distribution between neighbouring $\beta$-carbon atoms in EK from all-atom and coarse-grained simulations. The y-axis shows the normalized probability density, and the x-axis the inter-$\beta$-carbon distance in Å. Curves corresponding to the all-atom (AA) and coarse-grained (CG) simulations are shown.

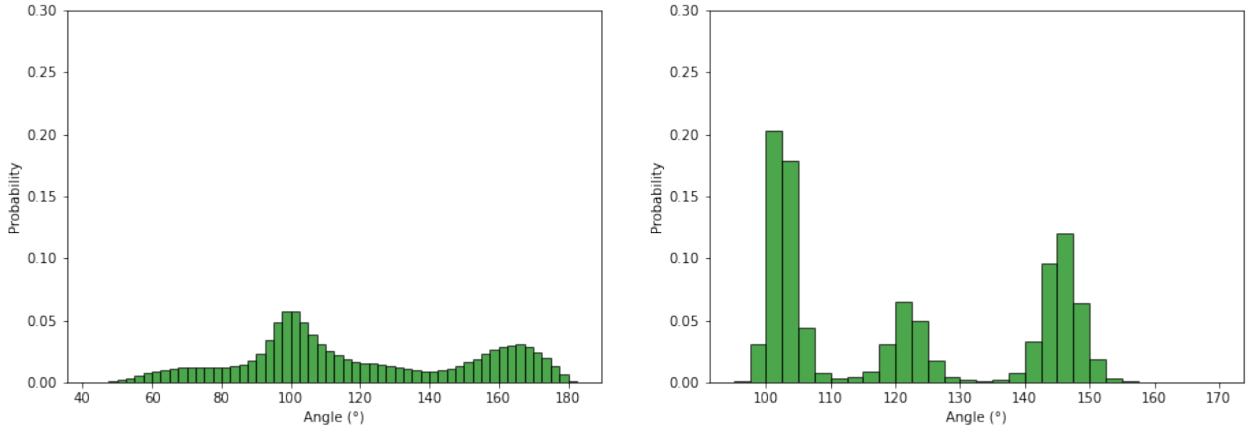


(a) All-atom angle distribution of EK

(b) Coarse-grained angle distribution of EK

Figure S2: Comparison of the angle distributions defined by each triad neighbouring C$\beta$ atoms in EK. (a) panel shows data for all-atom simulations, (b) panel for coarse-grained simulations. The y-axis shows normalized probability density, and the x-axis shows the angles measured between the carbon atoms in degree.

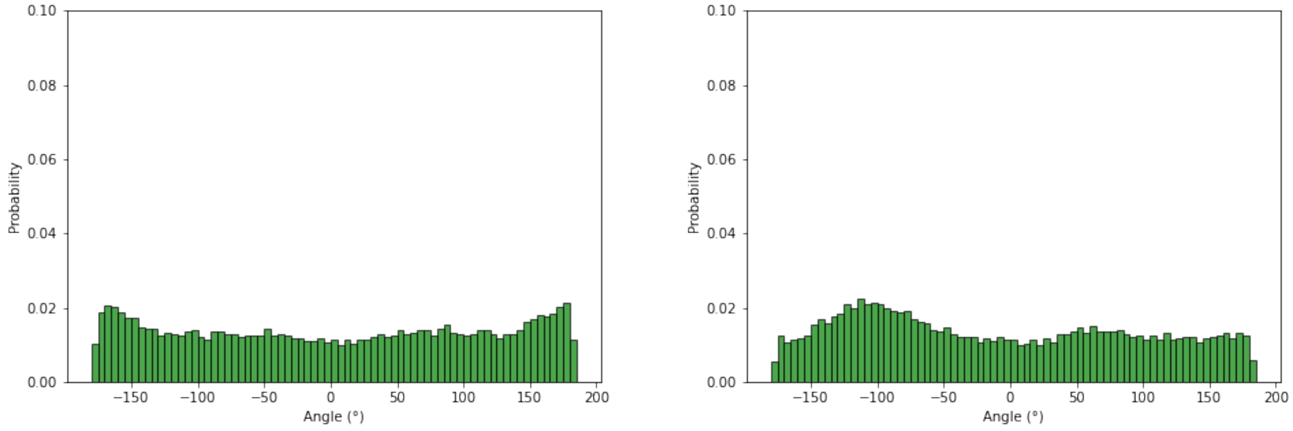


(a) All-atom dihedral angle distribution of EK

(b) Coarse-grained dihedral angle distribution of EK

Figure S3: Comparison of the dihedral angle distributions defined by each quartet of neighbouring C$\beta$ atoms in EK. (a) panel shows data for all-atom simulations, (b) panel for coarse-grained simulations. The y-axis shows normalized probability density, and the x-axis shows the dihedral angles measured between the carbon atoms in degree.

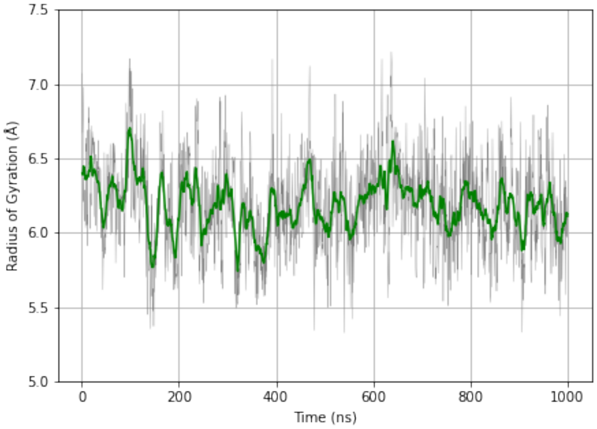


(a) Radius of gyration - all-atom for EK

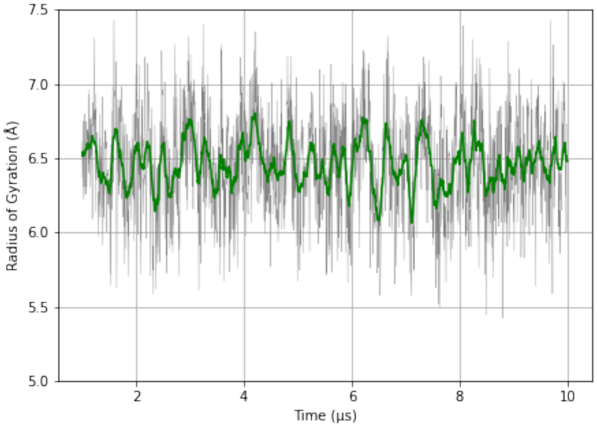


(b) Radius of gyration - coarse-grained for EK

Figure S4: Radius of gyration values of EK in all-atom and coarse-grained simulations. (a) panel shows data for all-atom simulations, (b) panel for coarse-grained simulations. The y-axis shows the radius of gyration values in Å, and the x-axis shows the time of the simulations.

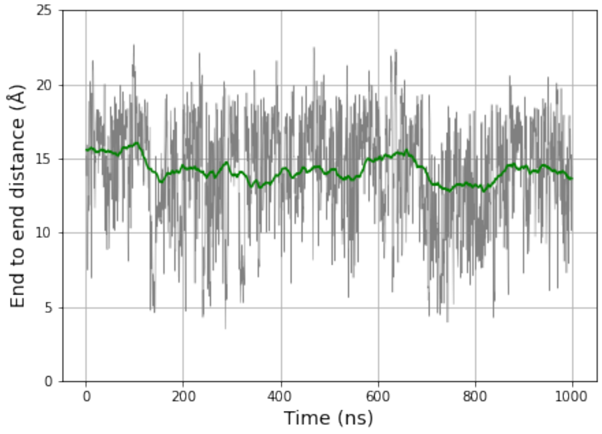


(a) All-atom end-to-end distance changes during simulation time for EK

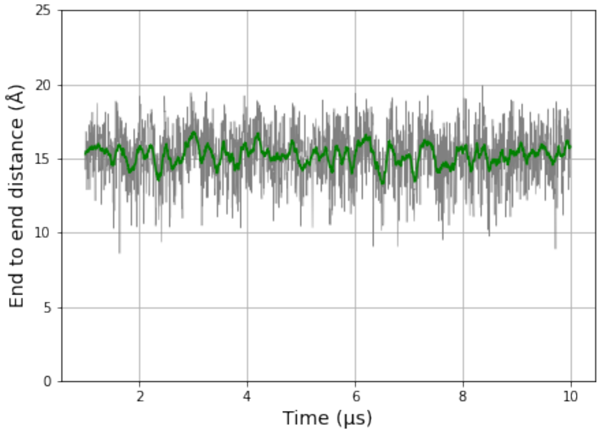


(b) Change in coarse-grained end-to-end distance over simulation time for EK.

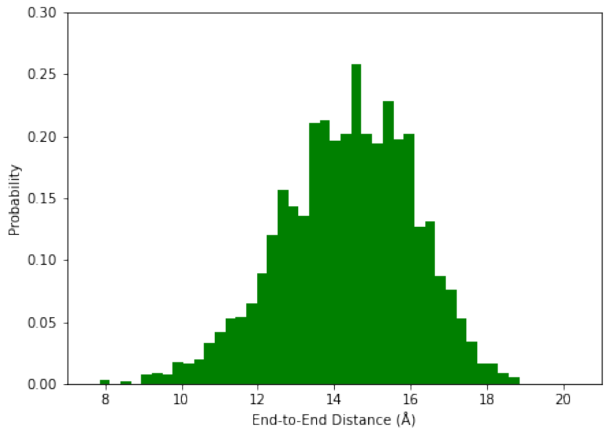


(c) Histogram of end-to-end distances from the all-atom simulations of EK.

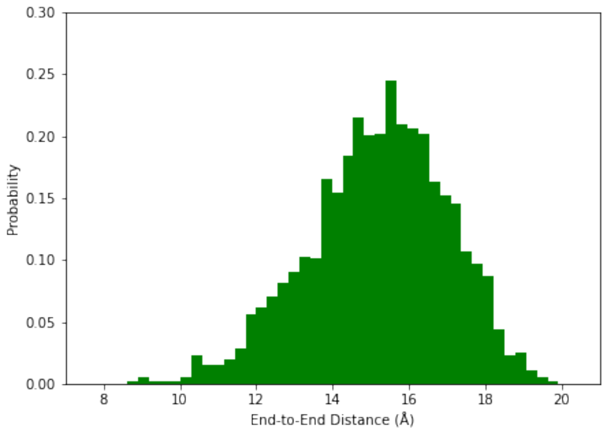


(d) Histogram of end-to-end distances from the coarse-grained simulations of EK.

Figure S5: End-to-end distances comparison for EK in all-atom and coarse-grained simulations. (a) panel shows the change of end-to-end distances in respect to time for all-atom simulations, (b) panel for coarse-grained simulations, (c) panel shows the normalized probability distribution of end-to-end distances for all-atom simulations, (d) panel for coarse-grained simulations. The end-to-end distance was defined as the distance between the first and last $\beta$-carbon atoms of the peptide. The y-axis shows the end-to-end distance values in Å, the x-axis shows the time of the simulations for panels (a), (b). The y-axis shows the normalized probability density, the x-axis shows the end-to-end distance distribution values in Å for panels (c), (d).
